# Chemical and Electrophysiological Characterisation of Headspace Volatiles from Yeasts Attractive to *Drosophila suzukii*

**DOI:** 10.1007/s10886-024-01494-x

**Published:** 2024-05-01

**Authors:** Irene Castellan, Claire Duménil, Guillermo Rehermann, Daniela Eisenstecken, Flavia Bianchi, Peter Robatscher, Urban Spitaler, Riccardo Favaro, Silvia Schmidt, Paul G. Becher, Sergio Angeli

**Affiliations:** 1 Faculty of Agricultural, Environmental and Food Sciences, Free University of Bozen-Bolzano, Bolzano, Italy; 2 Department of Plant Protection Biology, Chemical Ecology Horticulture Unit, Swedish University of Agricultural Sciences, Alnarp, Sweden; 3 Laboratory for Flavours and Metabolites, Institute for Agricultural Chemistry and Food Quality, Laimburg Research Centre, Auer-Ora, Italy; 4 Entomology Group, Institute for Plant Health, Laimburg Research Centre, Auer-Ora, Italy; 5 Competence Centre for Plant Health, Free University of Bozen-Bolzano, Bolzano, Italy

**Keywords:** Spotted wing drosophila, Yeast lure, *Saccharomycopsis vini*, *Hanseniaspora uvarum*, Headspace collection, Electroantennography

## Abstract

Chemical control of *Drosophila suzukii* (Diptera: Drosophilidae) based on the use of insecticides is particularly challenging as the insect attacks ripening fruits shortly before harvest. An alternative strategy may rely on the use of yeasts as phagostimulants and baits, applied on canopy as attract-and-kill formulations. The aim of this research was to identify the most attractive among six yeast species for *D. suzukii*: *Saccharomyces cerevisiae*, *Hanseniaspora uvarum*, *Clavispora santaluciae*, *Saccharomycopsis vini*, *Issatchenkia terricola*, and *Metschnikowia pulcherrima*. The volatile profile of *C*. *santaluciae* was described for the first time. Behavioural experiments identified *H. uvarum* and *S. vini* as the most attractive yeasts. The characterization of yeast headspace volatiles using direct headspace (DHS) and solid-phase microextraction (SPME) revealed several strain-specific compounds. With DHS injection, 19 volatiles were characterised, while SPME revealed 71 compounds constituting the yeast headspace. Both analyses revealed terpenoids including β-ocimene, citronellol, (*Z*)-geraniol (nerol), and geranial as distinct constituents of *S. vini*. *H. uvarum* and *S. vini* were further investigated using closed-loop stripping analysis (CSLA) and electroantennography. Out of 14 compounds quantified by CSLA, ethyl acetate, isoamyl acetate, β-myrcene, benzaldehyde and linalool were detected by *D. suzukii* antennae and might generate the strong attractiveness of *S. vini* and *H. uvarum.* Our results highlight a strong attraction of *D. suzukii* to various yeasts associated with both the flies and their habitat and demonstrate how different sampling methods can impact the results of volatile compound characterization. It remains to be demonstrated whether the distinct attraction is based on special adaptations to certain yeasts and to what extent the metabolites causing attraction are interchangeable.

**Supplementary Information:**

The online version contains supplementary material available at 10.1007/s10886-024-01494-x.

## Introduction

*Drosophila suzukii* (Matsumura) (Diptera: Drosophilidae) is a polyphagous invasive species responsible for major economic damage in stone fruits and soft fruits including grapevine berries. In only a few years, this insect has invaded several countries from its original Asian distribution and has established populations in many important agricultural regions around the world (Hauser 2011; Cini et al. 2012; Ometto et al. 2013; Benito et al. 2016; Kwadha et al. 2021). Females attack soft-skinned fruits of cultivated and wild plants by laying eggs under the skin of ripening fruits rendering them unmarketable (Walsh et al. 2011; Rombaut et al. 2017; Cai et al. 2019). Many high value crops such as blackberry, blueberry, strawberry, raspberry, cherry, plum, and grape are concerned (Lee et al. 2011; Ioriatti et al. 2015; Klick et al. 2016; Mazzi et al. 2017; Knapp et al. 2021). Presently, the most efficient viable tool for reducing economic losses caused by *D. suzukii* is the application of insecticides and physical barriers such as exclusion nets (Haye et al. 2016; Del Fava et al. 2017; Farnsworth et al. 2017; Shawer et al. 2018). Since fruit infestation can happen shortly before harvest, management is particularly challenging, and the use of insecticides prohibited during the preharvest interval. In addition, stricter measures are being imposed on the use of plant protection products hereby reducing the variety of chemicals available for pest control (Marchand 2023). The intense exposition to a limited number of registered products increases the risks of resistant populations to grow (Gress and Zalom 2019; Blouquy et al. 2021). There is an urgent need of developing more sustainable approaches for the control of *D. suzukii* with the aim of decreasing synthetic pesticide use (Tait et al. 2021). In the last few years chemo-ecological research in attractive volatiles, which are important for localization of food sources, mating- and egg-laying sites, has led to the development of new control methods against *D. suzukii* (Hamby and Becher 2016; Cloonan et al. 2018; Kienzle et al. 2020; Rehermann et al. 2022; Spitaler et al. 2022; Urbaneja-Bernat et al. 2022). *Drosophila suzukii* has the ability to lay eggs in ripening fruits, an adaptation shared with just a few members of the *Drosophila* genus like *Drosophila subpulchrella* (Atallah et al. 2014; Karageorgi et al. 2017; Crava et al. 2020). Indeed, many drosophilids show preference to lay eggs in overripe or fermenting fruits as it is well demonstrated for the model organism, *Drosophila melanogaster* (Becher et al. 2012; Kim et al. 2023). *Drosophila suzukii* exhibits a marked preference to ripening fruits (Abraham et al. 2015; Lee et al. 2016; Urbaneja‑Bernat et al. 2021; Abraham et al. 2022). Epiphytic yeast communities on these fruits contribute to specific aromatic profiles, enhancing the fruit attractivity during ripening (Jones et al. 2022). These yeasts were shown to be attractive to *D. suzukii* also when cultivated in growth media (Scheidler et al. 2015; Lasa et al. 2019; Bianchi et al. 2020a; Kleman et al. 2022; Rehermann et al. 2022). Several yeast species have been isolated from *D. suzukii* larvae and adults that had been feeding on fruits as well as from feeding galleries in infested fruit, the most common yeast species found being *Hanseniaspora uvarum* (Hamby et al. 2012; Bellutti et al. 2018; Lewis et al. 2019). Monitoring and other control measures like trapping strategies based on fermentation odours of mixtures of wine, apple cider vinegar, or rice vinegar have been evaluated in previous research (Landolt et al. 2012a, b; Iglesias et al. 2014; Ðurović et al. 2020). Volatiles of yeasts that are naturally associated with the flies are promising attractants for implementation in *D. suzukii* management.

To assign the attraction of *D. suzukii* to odours emitted by eight yeasts associated with the pest, our first goal was to identify the most attractive strains. A second goal was to characterise and to compare the volatile profiles of yeasts using different techniques of static and dynamic headspace analysis as direct headspace (DHS), solid-phase microextraction (SPME), and closed-loop stripping analysis (CLSA). Third, we wanted to identify antennally active volatile compounds of the most attractive yeasts which might be of prime importance for yeast attractiveness. The two most attractive yeasts, *Saccharomycopsis vini* and *H. uvarum* were then further studied to identify the specific antennally active volatiles, which might be of importance for yeast attractiveness.

## Methods and Materials

### Insects

*Drosophila suzukii* flies were reared at room temperature (23–25 °C) on a sugar-yeast-cornmeal diet (previously designated DSCD(a) with dry baker’s yeast (RUF Lebensmittelwerk KG, Quakenbrück, Germany) sprinkled over the surface (Bellutti et al. 2018), and maintained at 50–65% RH under a 12:12 h L: D (light: dark) photoperiod. The *D. suzukii* flies originated from infested grapes, cherries, blueberries, and blackberries collected in various locations in South Tyrol, Italy. The flies were collected in the same year as the experiments were conducted. Five to eight-day old adults were used in behavioural and electrophysiological experiments.

### Yeast Cultures

Eight yeasts were studied: *Hanseniaspora uvarum* 3.4, *Hanseniaspora uvarum* 2.2, *Hanseniaspora uvarum* 1.21, *Issatchenkia terricola* 2.1, *Metschnikowia pulcherrima* 3.2, *Saccharomycopsis vini* 1.33, *Clavispora santaluciae* 3.3, and *Saccharomyces cerevisiae* S288c (Table 1). The first seven have been isolated from feeding grooves of *D. suzukii* larvae in infested grapes in Italy in 2012 (Bellutti et al. 2018), while *S. cerevisiae* strain S288c is a widely used laboratory strain (NCBI Taxonomy ID: 559,292). Since *H. uvarum* was shown to be more attractive to *D. suzukii* compared to other yeasts (Scheidler et al. 2015), three strains of this species were included in our study. The three *H. uvarum* strains were isolated from feeding tunnels of *D. suzukii* larvae found in infested grapes originating from three different vineyards in South Tyrol (Bellutti et al. 2018). The strain *H. uvarum* 1.21 was isolated from grapes of the Lagrein variety, while *H. uvarum* 2.2 and *H. uvarum* 3.4 were isolated from the Vernatsch variety (alternative names: Schiava in Italy, Trollinger in Austria and Germany). *Clavispora santaluciae* is a yeast species recently classified by Drumonde-Neves et al. (2020) and was before mentioned in the literature as *Candida* sp. 3.3 (Bellutti et al. 2018; Bianchi et al. 2020b; Spitaler et al. 2020).

**Table 1:** The yeast strains used in this study

Yeast species	Strain	Accession number^a^	Order	Family
*Hanseniaspora uvarum*	LB-NB-1.21	KP298009	Saccharomycetales	Saccharomycodaceae
*Hanseniaspora uvarum*	LB-NB-2.2	MK567898	Saccharomycetales	Saccharomycodaceae
*Hanseniaspora uvarum*	LB-NB-3.4	MK567905	Saccharomycetales	Saccharomycodaceae
*Issatchenkia/Picchia terricola*	LB-NB-2.1	MK567903	Saccharomycetales	Pichiaceae
*Metschnikowia pulcherrima*	LB-NB-3.2	KP298012	Saccharomycetales	Metschnikowiaceae
*Saccharomycopsis vini*	LB-NB-1.33	KP298011	Saccharomycetales	Saccharomycopsidaceae
*Clavispora santaluciae*	LB-NB-3.3	KP298013	Saccharomycetales	Metschnikowiaceae
*Saccharomyces cerevisiae*	S288c		Saccharomycetales	Saccharomycetaceae

The first seven have been isolated from feeding grooves of *Drosophila suzukii* larvae in infested grapes (Bellutti et al. 2018), while *Saccharomices cerevisiae* strain S288c is a conventional laboratory strain

^a^The accession numbers were deposited in the GenBank NCBI

Yeast cultures were grown in 220 mL potato dextrose broth (PDB, 4 g/L potato starch, 20 g/L dextrose, Difco™, Becton Dickinson) at 25 °C for 30 h in a 250-mL Erlenmeyer flask closed with cotton and aluminium foil on a rotary shaker at 120 rpm. The inoculum (1 mL) was prepared with a loop full of yeast cells cultivated on potato dextrose agar (PDA, 4 g/L potato starch, 20 g/L dextrose, 15 g/L agar, Difco™, Becton Dickinson), which were then transferred in a 2-mL Eppendorf tube filled with 1 mL PDB and vortexed for 10 s at 1800 rpm. Preliminary trials showed that after 30 h all yeast cultures had reached the stationary phase. Six replicates of the inoculum were prepared for each yeast and an Erlenmeyer flask with non-inoculated PDB was kept under the same conditions as the yeasts for 30 h. Growth parameters of each yeast strain have been reported in Spitaler et al. (2020) for yeast fermentation broths prepared under similar conditions. Culture samples have been collected in aliquots of 8 mL in 20-mL clear glass vials (Sigma-Aldrich, St. Louis, MO) for DHS analysis, aliquots of 100 mL in 250-mL Pyrex® glass bottles (Pyrex, Charleroi, PA) for CLSA, aliquots of 5 mL of each sample were collected in 20-mL clear glass vials for SPME analysis, and aliquots of 2 mL were collected in 2-mL GC glass vials for behavioural analyses. Samples of PDB medium were collected for control. For storage, the fresh yeast cultures were filled in the respective glass vials, sealed, and stored in a freezer at -80 °C. The sealed glass vials were thawed at room temperature before use for analyses. PDB was used as growth medium because it was described as food source supporting the development of *D. suzukii*, is a feeding stimulant for *D. suzukii*, and no negative effects on *D. suzukii* were observed in earlier studies (Spitaler et al. 2020; Bianchi et al. 2020a; Kleman et al. 2022; Rehermann et al. 2022).

### Behavioural Assays

A 2-choice set-up with slight modifications compared to the assay described by Ruebenbauer et al. (2008) was used to evaluate the odour attractiveness of the different yeasts. For shipment to the behavioural laboratory, the 2 mL aliquots of the yeast cultures were freeze dried following the instructions reported by Schoug et al. (2006). For behavioural testing the samples were revitalized by adding 2 mL of PDB and 24 h later the live cultures were assayed. Round glass dishes (diam: 115 mm, height: 64 mm) closed with thin mesh were used for the behavioural assays. Each dish contained a small trap with a 2 mL revitalized yeast sample (treatment) and a trap with PDB medium (control). The traps were made of 4-mL glass vials that were closed with cut pipette tips that allowed the flies to get into the vials but prevented them from leaving.

Mated *D. suzukii* females were used to test for attraction to the individual yeasts. For obtaining mated females, newly emerged flies of both sexes were kept together for 4–5 days after emergence with an excess ratio of males : females (1.2–1.5 : 1). For sexing, flies were shortly anesthetized with CO_2_. Subsequently, female flies were kept in groups of 20 in rearing vials with wet cotton and sugar solution (5%) for 24 h, until the start of the experiment. Females in groups of 20 were placed into the dishes containing the different yeast treatments (*n* = 6). The number of females trapped in each treatment was scored after 1, 2, 3, 4, 5, 6, 20, and 24 h.

### Headspace Analysis by Direct Headspace (DHS)

Yeast samples were thawed and incubated at 80 °C for 30 min with the sample agitator alternating on/off for 25 s/5 s. The agitator speed was set at 250 rpm. With a 2.5 mL-HS syringe (Gerstel Inc.), 1 mL of headspace-collected air was injected (Gerstel MultiPurpose Sampler MPS, Gerstel Inc.) into a 7890 A Gas Chromatograph (GC) fitted with a non-polar HP-5MS column (30 m × 0.25 mm ID, 0.25 μm film thickness) and coupled with a 5975 C Mass Spectrometer (MS) (Agilent Technologies) in split mode (split ratio 3:1) at 200 °C injection port temperature. For GC, helium was used as carrier gas at a flow rate of 1.2 mL/min and a velocity of 39.92 cm/s. The starting temperature of 35 °C was held for 4 min, followed by an increase of 7.5 °C/min until a temperature of 200 °C was reached then increased again at 40 °C/min until a temperature of 250 °C was reached and held for 2 min. The mass spectrometric data were acquired in full scan mode over a range of *m/z* 34–300 at a rate of 2.72 spectra/s. Data were processed and analysed using the ChemStation software (Agilent Technologies). Compounds were tentatively identified by comparing their mass spectra with those in the reference libraries NIST 14 (Gaithersburg) and ‘Wiley7N’ (Wiley). Identities of compounds were verified by comparison of linear retention indexes (LRI), and prominent and typical ion fragments of authentic standard compounds (Sigma-Aldrich).

### Headspace Analysis by Solid-Phase Micro Extraction (SPME)

Headspace volatiles released by the yeast strains were also identified using SPME in order to detect chemicals that were not picked up by DHS. Samples were thawed and held in the Gerstel Autosampler (Gerstel GmbH & Co. KG) at 15 °C, incubated at 30 °C for 15 min and extracted with a CAR/DVB/PDMS fiber 50/30 µm for 30 min. The analysis was done on a gas chromatograph-time of flight-mass spectrometry (GC-TOF-MS) using an Agilent 7890B GC instrument (Agilent Technologies) coupled to a LECO Pegasus GC-HRT-MS instrument (Leco Corperation) controlled with ChromaTOF software 5.32. Sample analysis was randomized and a pooled sample as well as a blank sample were injected after every 10 samples for quality control and to perform a background subtraction of column deterioration/background/degradation peaks during the data pre-processing steps. Desorption was performed at 250 °C for 2 min. The GC instrument was operated in splitless mode using helium as carrier gas with 1 mL/min and the separation was achieved on a ZB-WAX column (60 m x 0.25 mm ID, 0.25 μm thickness, Phenomenex). A gradient temperature programming of 35 °C (5 min), 35–245 °C linear (5.5 °C/min), 245 °C (15 min) was used. Transfer line temperature and ion source temperature were set at 250 °C; ion source voltage was 70 eV. The mass spectrometric data were acquired in full scan mode over a range of *m/z* 40–510 at a rate of 3.4 spectra/s. The analytical standard perfluorotributylamine (PFTBA) was used as calibrant for maintaining mass accuracy. In the ChromaTOF software, the settings of parameters for target analyte finding were the following: data points for averaging at auto; peak width at 2, mass tolerance at 20 ppm, the base peak was used for quantitation of peak height and one to two qualifier ions were selected. The target analytes list was compiled by comparison of the mass fragmentation patterns of individual component with commercially available NIST 11 mass spectral library. Metabolites were annotated based on the spectrum similarity and LRI from published records (PubChem, NIST, Pherobase).

### Headspace Analysis by Closed-Loop Stripping Analysis (CLSA)

Headspace volatiles of the two most attractive yeasts (*S. vini* and *H. uvarum* 2.2) were further characterized by air entrainment with CLSA filters. CLSA filters consisting of 1.5 mg activated charcoal (LR-type, Brechbühler AG) were fitted into the plastic lid of a 250-mL glass bottle containing 100 mL of thawed yeast culture. The system was connected to a Pye Air Entrainment Kit® (BJ Pye, Kings Walden, UK). Filtered clean air was pumped through an inlet port made of PTFE tubing at a rate of 1,000 mL/min into the bottle. Air was pulled out through the CLSA filter using a short Teflon tube at a rate of 400 mL/min. The output flow was less than the input rate ensuring that unfiltered air was not drawn into the collection bottle from outside. Collections lasted 3 h. Each CLSA filter was then eluted with 100 µL of GC-grade dichloromethane solvent (Sigma-Aldrich) into 1.1-mL glass vials (Sigma-Aldrich) hereby collecting all volatiles captured by the charcoal. Extracts were stored at -80 °C until use in GC-MS. After elution, filters were washed with solvents of different polarity: GC-grade dichloromethane, HPLC-grade methanol, and HPLC-grade heptane (Sigma-Aldrich) and dried 10 min at 60 °C. Two µL of extracts were injected in a splitless mode GC-MS (as above) when the inlet valve was at 280 °C. Helium was used as a carrier gas. The starting temperature of 50 °C was held for 1.5 min, followed by an increase of 7.5 °C/min until a temperature of 250 °C was reached and then held for 10 min. The mass spectrometric data were acquired in full scan mode over a range of *m/z* 35–400 at a rate of 3.89 spectra/s. Data acquisition and analysis were carried out as described above. Six replicates for *H. uvarum* 2.2, *S. vini*, and PDB were sampled. The quantification of identified compounds in the extracts was done by 5-point calibration of the standard compounds and evaluation of peak areas in the selected ion-monitoring mode.

### Electroantennography (EAG) and Gas Chromatography-Electroantennography Detection (GC-EAD)

To determine whether compounds identified from headspace extracts are detected by adult *D. suzukii*, EAG and GC-EAD experiments were performed. EAG experiments permitted to measure the antennal responses to volatile yeast compounds. GC-EAD allowed to elute each pure chemical on the antenna and eliminate possible antennally active contaminants from the standard solutions (described below). A fly was immobilized in a truncated plastic pipette tip with half of its head protruding from the narrow end. The antennal activity was measured by placing a recording electrode over the tip of the antenna and an indifferent electrode at the base of the antenna within the head capsule. Ag–AgCl glass electrodes were filled with ringer solution (Beadle–Ephrussi Ringer solution of NaCl, KCl, CaCl_2_, composition as in Matheu et al. 2011). Signals were passed through a high input impedance amplifier (2-channel USB acquisition controller, IDAC-2; Syntech) and analysed using GC-EAD 2014 v1.2-5 (Syntech). Three males and three females were recorded in each experiment. In EAG, a 1.5 s air pulse (odour stimulus) was given through a glass Pasteur pipette (Thermo Fisher Scientific, USA) containing an aliquot of 30 µL of a solution of a chemical in paraffin oil on a filter paper (15 mm diameter, Whatman grade 1, USA) placed within the larger end and closed with a 1-mL pipette tip, into a carbon-filtered humidified air stream directed at the fly preparation. In GC-EAD, the electroantennography detector was coupled with a GC (7820 A, Agilent Technologies) equipped with a Flame Ionization Detector (FID). The fly was prepared and connected as described above. Three µL of sample were injected in the GC column (HP-5MS Agilent 19,091 J-413 column, 0.25 μm coating 30 m length and 0.32 mm diameter) through a Cool-On-Column (COC) injector. Helium at a flow rate of 2.5 mL/min was used as carrier gas. The oven method was programmed as follows: inject at 50 °C and hold for 1.8 min, then 7.3 °C/min until 250 °C and hold for 3 min. The temperature of the injector was 250 °C and detector temperature was set at 350 °C. The column effluent was mixed with a nitrogen make-up and split at a 1:1 ratio, one part flowing to the FID, the other going through a transfer line (170 °C) (Syntech) into a charcoal-filtered and humidified airstream channelled to the mounted antennal preparation. The signal was amplified via an EAG amplifier (as above). Authentic standards of 14 compounds characterized in the CLSA analysis from the headspace collection of *H. uvarum* 2.2 and *S. vini* were tested in EAG; the standard of β-ocimene contained a mixture of the (*E*) and (*Z*) isomers. In addition, ethyl acetate and ethanol were tested as these compounds were characterized in considerable amounts from the DHS analysis previously conducted. 2-Heptanone was introduced as a positive control as it is known to be antennally active (Dobritsa et al. 2003; Keesey et al. 2015; Lebreton et al. 2017) but was initially absent in the yeast volatiles. All standards were tested as 10^−3^ dilutions in paraffin oil. A control cartridge containing only paraffin oil was used on each fly. Standards for which a response was significantly higher than for paraffin oil were considered antennally active following a Wilcoxon sum-ranked test.

Then, a solution of all 17 authentic standard compounds (18, counting *Z*- and *E*-β-ocimene separately) was created as an isomix, meaning that equal amounts of each standard were mixed and diluted in dichloromethane (10^−3^) to be tested with GC-EAD. In addition, the headspace (5 µL) of a mixture of neat standards of ethanol and 2-heptanone was injected directly into the GC-EAD in order to locate ethanol which was masked by the co-eluting dichloromethane. All GC peaks that consistently elicited an antennal response of at least two out of six flies were considered active. Each standard GC peak was confirmed by the respective LRI.

### Statistical Analysis

All statistical analyses were performed with the software R (R Core Team 2020). A generalized linear model (GLM) fitted with a Poisson distribution was used to evaluate female attraction to yeast samples. To analyse trapping between the different yeast along time a mixed-effect generalized linear model (GLMM) fitted with a negative binomial distribution and replicates as a random factor, was performed (MASS package, Venables and Ripley 2003; lme4 package, Bates et al. 2015) followed by a Tukey pairwise comparison (multcomp package, Hothorn et al. 2008). For data visualization, tidyverse was used (Wickham et al. 2019). Variation in the volatile profiles of the yeast strains was evaluated with one-way ANOVA followed by *posthoc* Tukey test and unpaired t-test. Antennal responses to compounds were compared to the response to a control stimulus using a paired t-test. Male and female responses were compared with a Wilcoxon sum-ranked test. For volatile profiles and antennal responses, the distribution of data was assessed using Shapiro-Wilk Normality test and the statistical tests were chosen accordingly. The dataset of 71 annotated compounds from the SPME analysis was normalized by log transformation, replacing missing values (expressed as N.D. in Electronic Supplementary Table 1) by 0.01 for each compound under investigation, followed by auto scaling. The heatmap was generated using pheatmap package (Kolde 2019) and the principal component analysis (PCA) was performed using Metaboanalyst (Chong et al. 2018).

## Results

**Fig. 1:**
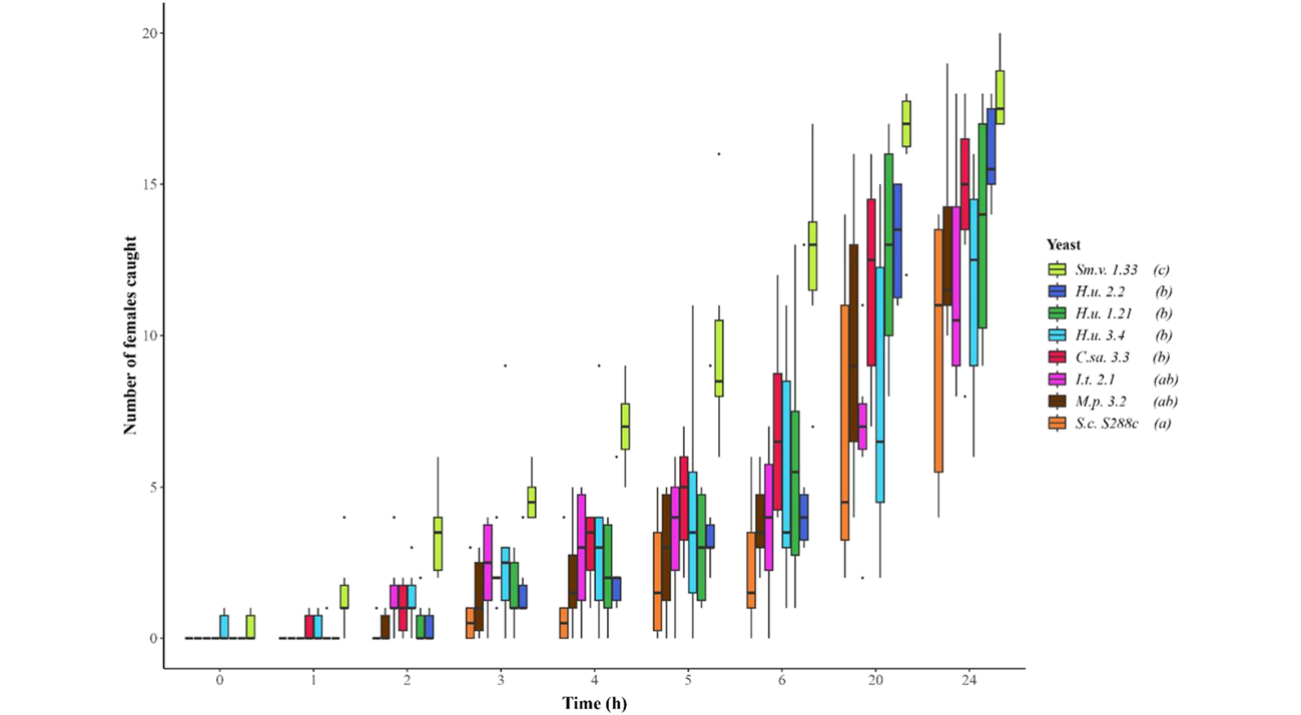
Temporal catches of *Drosophila suzukii* females trapped by the individual yeasts in Potato Dextrose Broth after 1, 2, 3, 4, 5, 6, 20 and 24 h. The first measurement (0 h) was taken within 5 min after starting the test. Coloured boxplots represent the catches for each yeast sp. (*n* = 20 females x 6 replicates x yeast species). A black line within the box marks the median, dots represent outliers, and whiskers represent the data within 1.5 x the interquartile range. Following yeast names, different letters mean significant differences comparing the number of flies caught along the experimental time between yeasts (GLMM-Tukey contrast; *p* < 0.05). *H.u., Hanseniaspora uvarum; S.c., Saccharomyces cerevisiae; I.t., Issatchenkia terricola; C.sa., Clavispora santaluciae; S.v., Saccharomycopsis vini; M.p., Metschnikowia pulcherrima*

The yeasts *S. vini* and *H. uvarum* 2.2 are highly attractive. The eight yeasts attracted a significant higher number of female *D. suzukii* flies over 24 h compared to PDB (GLM; *p* < 0.05) (Electronic Supplementary Fig. 1). Overall, *S. vini* was outstanding as the most attractive yeast, while baker’s yeast *S. cerevisiae* was distinct in being the least attractive (GLMM-Tukey contrast; *p* < 0.05) (Fig. 1). In total, 90% of the flies tested for attraction to *S. vini* got attracted within 24 h while *S. cerevisiae* attracted 48% of the flies (Electronically Supplementary Fig. 1). Two yeasts, *S. vini* and *H. uvarum* 3.4, lured the first females during the first hour of the experiments into the traps. Over the whole 24 h period the three strains of *H. uvarum* tested did not differ in their capacity to attract flies (GLMM-Tukey contrast; *p* < 0.05) (Fig. 1), while *H. uvarum* 2.2 attracted the highest absolute number of flies. We therefore selected *H. uvarum* 2.2 for further investigation in addition to *S. vini*. All the other yeasts showed intermediate attractiveness to *D. suzukii* (Fig. 1).

### Yeasts Release Distinct Volatile Profiles in DHS and SPME

Each yeast released a distinct volatile profile according to both DHS and SPME techniques. Using direct headspace sampling followed by gas chromatography coupled to mass spectrometry (DHS-GC-MS) for identification and quantification, nineteen volatile compounds were identified and annotated for the eight selected yeast cultures (Table 2) and quantitative differences among headspace components were confirmed by one-way ANOVA. The compounds could be classified into four acetates, four alcohols, one acid, one aldehyde, five esters and four terpenes (Table 2). Of the various compounds identified in the yeasts, only ethanol, 3-methylbutan-1-ol, and 2-phenylethanol were common to all. Ethanol was particularly abundant in *M. pulcherrima*. 2-phenylethanol was detected in every yeast, but often only in trace amounts. Ethyl acetate was found in all headspaces in large amounts, excluding *I. terricola* and *S. cerevisiae*. The only acid detected (octanoic acid) was found in traces in *S. cerevisiae* only. Benzaldehyde was detected in traces in *S. vini* and in all the *H. uvarum* strains.

**Table 2:** Volatile organic compounds from yeast strain headspaces identified by DHS-GC-MS

Compound	LRI^A^	VOC average amount in TIC^B^																					
		C. sa. 3.3	M. p. 3.2					S. v. 1.33		I. t. 2.1			S. c. S288			H. u. 1.21			H. u. 2.2			H. u. 3.4	
Acetates																							
Ethyl acetate	618	208.5 ± 79.9^c^	22.7 ± 6.3^a^					78.6 ± 31.9^b^		nd			nd			65.9 ± 27.5^b^			30.8 ± 16.9^b^			91.6 ± 24.5^b^	
Propyl acetate	707	1.1 ± 0.9	nd					nd		nd			nd			nd			nd			nd	
2-Methylpropyl acetate	772	1.3 ± 0.8	nd					nd		nd			nd			nd			nd			nd	
Isoamyl acetate	879	23.8 ± 9.6^a^	nd					nd		nd			nd			0.9 ± 0.5^b^			0.4 ± 0.2^b^			0.8 ± 0.4^b^	
Alcohols																							
Ethanol	< 600	61.3 ± 33.2^ab^	130.4 ± 24.8^b^					39.7 ± 6.7^a^		59.7 ± 30.3^ab^			104.5 ± 100.7^ab^			37.8 ± 18.9^a^			30.4 ± 12.7^a^			40.5 ± 28.6^a^	
2-Methylpropan-1-ol	626	nd	nd					nd		3.3 ± 1.5^b^			1.6 ± 1.5^a^			nd			nd			nd	
3-Methylbutan-1-ol	728	2.8 ± 1.7^a^	5.3 ± 2.1^ab^					5.4 ± 2.4^ab^		8.3 ± 4.4^b^			9.6 ± 7.1^b^			1.3 ± 1.2^a^			0.5 ± 0.8^a^			0.4 ± 0.5^a^	
2-Phenylethanol	1115	traces	0.4 ± 0.1^b^					traces		0.3 ± 0.2^b^			0.2 ± 0.1^a^			traces			traces			traces	
Acid																							
Octanoic acid	1171	nd	nd					nd		nd			traces			nd			nd			nd	
Aldehyde																							
Benzaldehyde	962	nd	nd					traces		nd			nd			traces			traces			traces	
Esters																							
Ethyl hexanoate	1001	nd	nd					nd		0.4 ± 0.2^b^			0.2 ± 0.1^a^			nd			nd			nd	
Ethyl octanoate	1198	nd	nd					nd		1.3 ± 0.4^b^			0.6 ± 0.5^a^			nd			nd			nd	
Ethyl nonanoate	1298	nd	nd					nd		0.1 ± 0.0			nd			nd			nd			nd	
Ethyl 9-decenoate	1389	nd	nd					nd		nd			0.1 ± 0.0			nd			nd			nd	
Ethyl decanoate	1396	nd	nd					nd		0.3 ± 0.1^a^			0.6 ± 0.5^a^			nd			nd			nd	
Terpenoids																							
(*E*)-β-Ocimene	1101	nd	nd					0.2 ± 0.1		nd			nd			nd			nd			nd	
Citronellol	1230	nd	nd					0.1 ± 0.0		nd			nd			nd			nd			nd	
(*E*)-Geraniol	1257	nd	nd					1.1 ± 0.6		nd			nd			nd			nd			nd	
Geranial	1268	nd	nd					traces		nd			nd			nd			nd			nd	

*H. u., Hanseniaspora uvarum; S. c., Saccharomyces cerevisiae; I. t., Issatchenkia terricola; C. sa., Clavispora santaluciae; S. v., Saccharomycopsis vini; M. p., Metschnikowia pulcherrima.* Numbers reported after the yeast species abbreviations indicate the strain number. Control samples of the growth medium Potato Dextrose Broth (PDB) did not contain any of the listed compounds. Average values (± SD, *n* = 6) are shown

^A^Linear Retention Index (LRI) on an apolar HP-5MS, calculated from experimental retention times, were verified with authentic standards and were similar to those from published libraries. ^B^The amount in TIC (Total Ion Chromatogram) of each compound in the yeast cultures is the mean peak area (mean ± standard deviation) of six replicates divided by 10^6^. ‘nd’, not detected; Unless stated otherwise, isomers were not identified. Significant differences between yeasts for each compound are indicated using different lowercase letters [ANOVA, Tukey test, *p* < 0.05]

The yeast *S. vini*, which was the most attractive to *D. suzukii* in behavioural experiments, released a unique headspace composition consisting mainly of ethyl acetate, alcohols (ethanol, 3-methylbutan-1-ol) and terpenes ((*E*)-β-ocimene, geranial, citronellol, (*E*)-geraniol. Furthermore, *S. vini* released the largest amounts of ethyl acetate after *C. santaluciae* and *H. uvarum* 3.4. In the least attractive yeasts *S. cerevisiae* and *I. terricola*, mostly other ethyl esters (ethyl hexanoate, ethyl octanoate, ethyl nonanoate, ethyl 9-decenoate, ethyl decanoate) were found, whereas no acetates were detected by DHS. On the other hand, a large number of acetates (ethyl acetate, propyl acetate, 2-methylpropyl acetate, 3-methylpropyl acetate) was found in the yeast *C. santaluciae*. Propyl acetate and 2-methylpropyl acetate were found exclusively in that yeast.

**Fig. 2:**
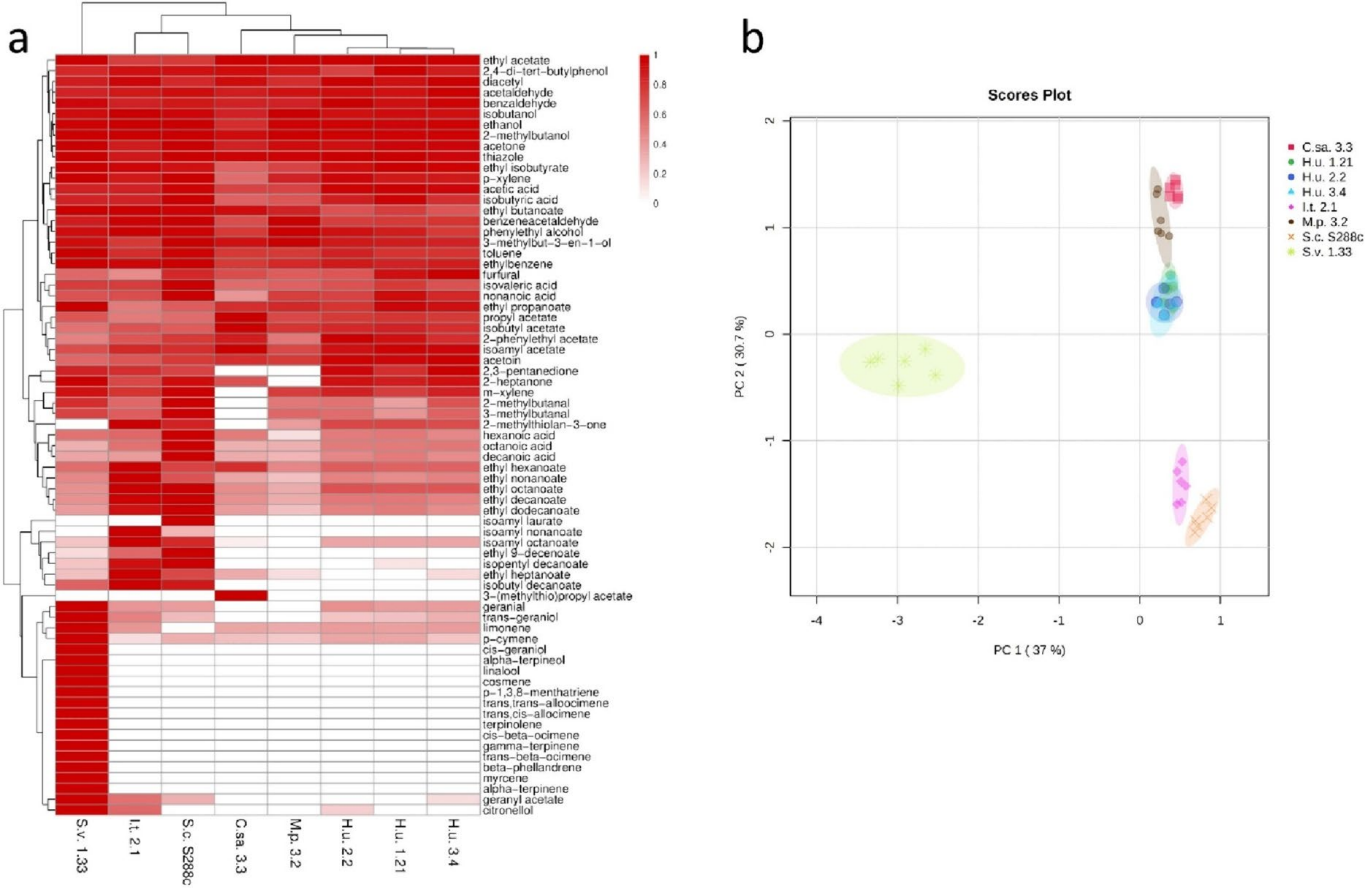
**a** Heatmap of the peak heights of annotated VOCs in the eight different yeasts using solid-phase microextraction followed by gas chromatography-time of flight-mass spectrometry (SPME-GC-TOF-MS). Average values for each yeast (*n* = 6) are shown. Variations within single compounds are displayed using a colour scale ranging from white (not detected) to dark red (highest value) as shown in the legend. Both yeasts and VOCs are hierarchically clustered. PDB data are available as Electronic Supplementary Table 1. **b** Two-dimensional score plot generated using the first two principal components of the PCA. The PCA was performed including all the VOCs annotated in the eight yeasts under investigation analysed through SPME-GC-TOF-MS. *H. u., Hanseniaspora uvarum; S. c., Saccharomyces cerevisiae; I. t., Issatchenkia terricola; C. sa., Clavispora santaluciae; S. v., Saccharomycopsis vini; M. p., Metschnikowia pulcherrima*

Solid phase microextraction followed by gas chromatograph-time of flight-mass spectrometry (SPME-GC-TOF-MS) analysis detected 71 compounds (Fig. 2A; Electronic Supplementary Table 1). The compound classes that were detected belong to acetates (six metabolites), acids (seven metabolites), alcohols (seven metabolites), aldehydes (six metabolites), esters (15 metabolites), hydrocarbons (five metabolites), ketones (six metabolites), and terpenes (19 metabolites). Five of the acetates were present in all yeasts, while 3-(methylthio)propyl acetate was detected in *C. santaluciae* only. The acid detected in highest amounts was acetic acid followed by isobutyric acid. Generally, acids were found to be higher in *S. cerevisiae* compared to the other yeasts and lowest in the growth medium alone (Electronic Supplementary Table 1). The alcohol detected in highest amounts was ethanol, being highest in *S. cerevisiae*. Thiazole and 2,4-di-*tert*-butylphenol were found to be higher in PDB compared to the yeasts. The aldehydes were observed mainly in PDB except for acetaldehyde, which was more abundant in all yeast samples. Out of the 15 esters, all were present in *S. cerevisiae* and 14 were present in *I. terricola* except isoamyl laurate. Hydrocarbons and ketones were detectable in almost all samples.

While all of the nineteen terpenoids (myrcene, α-terpinene, limonene, β-phellandrene, (E)-β-ocimene, γ-terpinene, (*Z*)-β-ocimene, terpinolene, (*E, Z*)-allocimene, (*E, E*)-alloocimene, p-1,3,8-menthatriene, cosmene ((3*E*,5*E*)-2,6-dimethyl-1,3,5,7-octatetraene), linalool, α-terpineol, geranial, geranyl acetate, citronellol, (*Z*)-geraniol (commonly known as nerol), (*E*)-geraniol) were found in *S. vini*, only a subset of them (limonene, geranial, citronellol, geranyl acetate, and (*E*)-geraniol) was also found in other yeasts (Fig. 2). Limonene and geranial were also present in the PDB growth medium, which could be an explanation for trace amounts detected in other yeasts (Electronic Supplementary Table 1). Geranyl acetate was observed in traces in *I. terricola* and *S. cerevisiae*, citronellol in *I. terricola*, and (*E*)-geraniol in the three *H. uvarum* strains, *I. terricola*, and *S. cerevisiae*. Those similarities and dissimilarities among the yeasts as well as the single volatile organic compounds (VOCs) were highlighted visually in a heatmap based on hierarchical clustering analysis (Fig. 2A). The hierarchical clustering analysis in the heatmap and the PCA (Fig. 2B) display the similarities among yeasts, showing that the three *H. uvarum* strains show a similar VOCs profile. *I. terricola* and *S. cerevisiae* showed a similar VOC profile as well as *C. santaluciae* and *M. pulcherrima*, while *S. vini* shows a clearly different profile compared to the other yeasts. The main differences of the *S. vini* profile can be explained by the presence of specific terpenoids, which are lacking in all other yeasts. The average values and standard deviations for each measured compound are displayed in Electronic Supplementary Table 1.

Volatiles of *S. vini* and *H. uvarum* 2.2 elicit physiological responses in *D. suzukii*. Volatile compounds from headspaces of *S. vini* and *H. uvarum* 2.2 were further identified and quantified using closed loop stripping analysis followed by gas chromatography coupled to mass spectrometry (CLSA-GC-MS), as those two strains were the most attractive yeasts to *D. suzukii* females, based on the behavioural assays. This technique permitted to detect and quantify specific compounds and to highlight further differences between the two yeasts (Table 3). As shown in Table 3, S. *vini* and *H. uvarum* 2.2 were characterised by distinct volatile profiles. In *S. vini* we detected nine compounds, six of which were yeast-specific monoterpenes: citronellol, (*E*)-geraniol, geranial, (*E*)-β-ocimene, linalool and β-myrcene. In *H. uvarum* 2.2 headspace we found eight compounds, two of which were yeast-specific acetates (isoamyl acetate and 2-phenylethyl acetate), two yeast-specific fatty acid esters (ethyl octanoate and ethyl decanoate), and one yeast-specific fatty acid (octanoic acid). Both yeasts released benzaldehyde, benzyl alcohol and 2-phenylethanol, but *H. uvarum* 2.2 released benzyl alcohol in significantly larger amounts compared to *S. vini* [unpaired t-test, t_6.77_ = 2.78, *p* = 0.028].

**Table 3:** Compounds identified in headspace extracts of *Saccharomycopsis vini (S.v.)* 1.33 and *Hanseniaspora uvarum (H.u.)* 2.2 via CLSA-GC-MS for 3 h

	LRI^A^		Mean (± SD) amount (µg/3 h)		
Compound			S. v. 1.33		H. u. 2.2
Acetates					
Isoamyl acetate	883		-		87.8 (± 45.7)
2-Phenylethyl acetate	1258		-		45.6 (± 9.6)
Aldehyde					
Benzaldehyde	961		392.6 (± 307.9)		644.9 (± 295.2)
Alcohols					
Benzyl alcohol	1034		17.5 (± 10)^a^		46.4 (± 23.5)^b^
2-Phenylethanol	1114		563.9 (± 111.7)		593.1 (± 199.3)
Acid					
Octanoic acid	1174		-		834.3 (± 432)
Esters					
Ethyl octanoate	1199		-		4.9 (± 1.2)
Ethyl decanoate	1398		-		1.3 (± 0.7)
Monoterpenoids					
β-Myrcene	992		98.1 (± 74.6)		-
(*E*)-β-Ocimene	1050		32.8 (± 22.7)		-
Linalool	1103		26.3 (± 6.3)		-
Citronellol	1229		136.9 (± 91.5)		-
(*E*)-Geraniol	1260		1605.2 (± 909.1)		-
Geranial	1274		66.6 (± 97.6)		-

Compounds were identified with GC-MS and confirmed with authentic standards. Retention indices were also compared with those from published chemical libraries, quantified from headspace extracts of standards collected by air entrainment and CLSA. Yeasts were cultured in Potato Dextrose Broth (PDB) medium. No compounds were detected in headspace extracts of PDB. Average values (± standard deviation) for each yeast extract (*n* = 6) are shown. Quantification was performed by 5-point calibration curve with authentic standard. Unless stated otherwise, standards were in racemic mixtures and of the highest purity available

^A^Linear Retention Index (LRI) on an apolar HP-5MS, calculated from experimental retention times, were similar to the standards’ and from published libraries. Numbers with different lowercase letters are significantly different [unpaired t-test, *p* < 0.05]

**Fig. 3:**
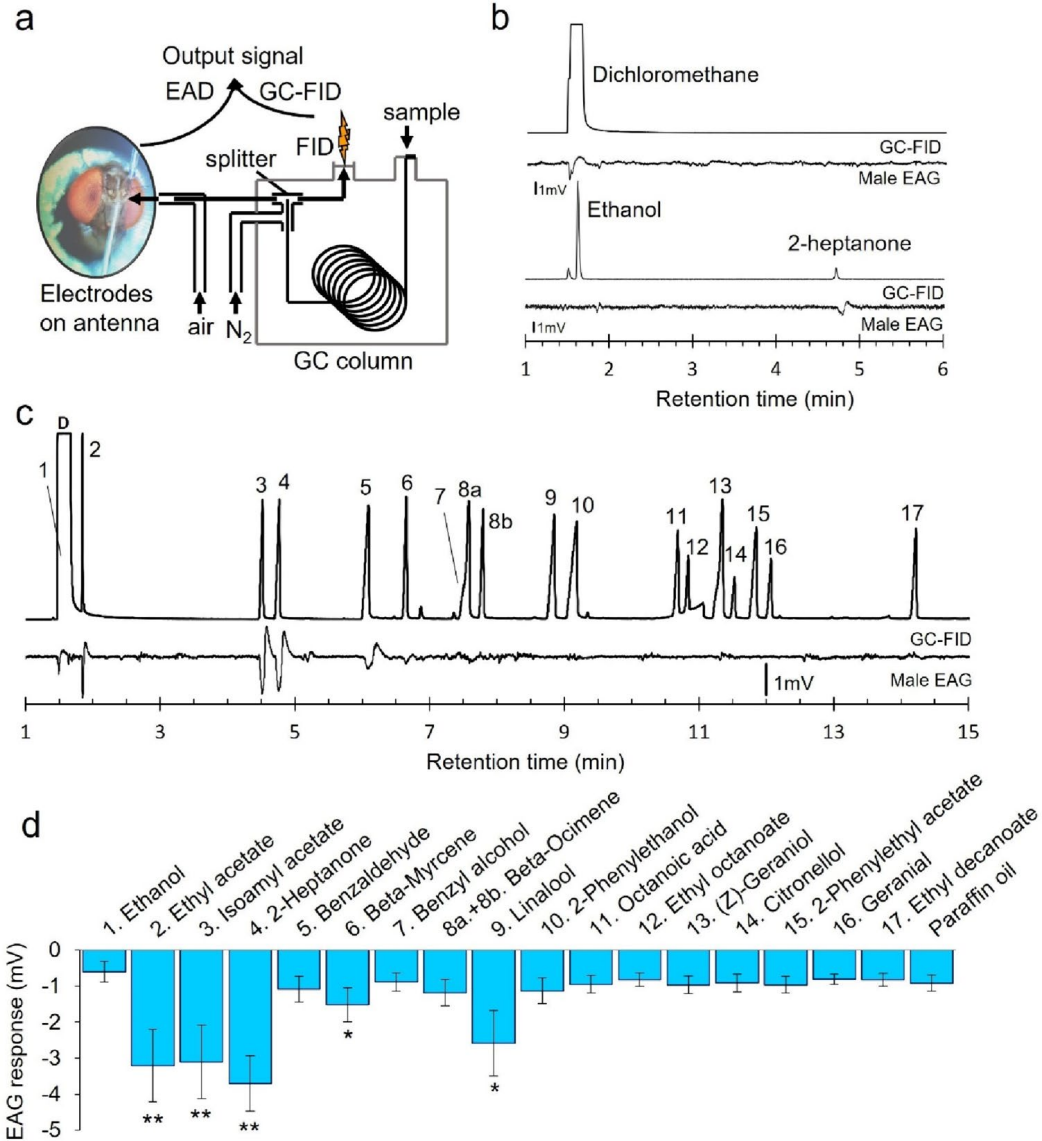
*Drosophila suzukii* antennal responses to the compounds identified from *Saccharomycopsis vini* 1.33 and *Hanseniaspora uvarum* 2.2 by closed-loop stripping analysis coupled with gas chromatography mass spectrometry (CLSA-GC-MS). Three males and three females were tested in all GC-EAD and EAG experiments. The EAG responses were pooled as they did not differ significantly between males and females (Wilcoxon rank sum exact test, *p* < 0.05). Representative traces are reported as follows. **a** Scheme of gas chromatography-electroantennography (GC-EAD) setup. **b** Males *D. suzukii* GC-EAD responses to headspace of dichloromethane solvent (upper traces), and a mixture of pure ethanol and 2-heptanone authentic standard (lower traces). **c** A male GC-EAD response to an authentic standard mixture of 10^−3^ dilution in dichloromethane is shown on the trace. Antennal responses for more than two flies were located at peak 2, 3, 4, 6, and 9. Peaks 7 and 8a were overlapping. Annotations: (1) Ethanol, (2) Ethyl acetate, (3) Isoamyl acetate, (4) 2-Heptanone, (5) Benzaldehyde, (6) β-Myrcene, (7) Benzyl alcohol, 8a. (*E*)-β-Ocimene, 8b. (*Z*)-β-Ocimene (co-occurring in the authentic standard of 8a, but not detected in the CLSA), 9. Linalool, 10. 2-Phenylethanol, 11. Octanoic acid, 12. Ethyl octanoate, 13. Citronellol, 14. 2-Phenylethyl acetate, 15. (*Z*)-Geraniol (also known as nerol), 16. Geranial, 17. Ethyl decanoate, D. Dichloromethane. **d** Mean (+/-SEM) amplitude of antennal response in EAG in response to an odour stimulus from standards of dilution 10^−3^ in paraffin oil compared to paraffin oil [paired t-test]: “**” *p* < 0.01, “*” *p* < 0.05. The standard 2-heptanone was used as a positive control. Statistical analyses can be accessed in Electronic Supplementary Table 2

Next, GC-EAD and EAG experiments were performed to identify which of these compounds from *S. vini* and *H. uvarum* 2.2 were detected by male and female *D. suzukii* (Fig. 3). According to the chromatograms in DHS analysis, ethyl acetate and ethanol emissions were both very low in the profile of *H. uvarum* 2.2, while ethyl acetate emission was fairly low in the profile of *S. vini*. Out of the standard mixture tested, six compounds were antennally active in GC-EAD when tested at a 10^−3^ dilution: ethyl acetate, isoamyl acetate, 2-heptanone, benzaldehyde, β-myrcene, and linalool (Fig. 3d). Unlike the others which gave an antennal response for all six flies tested in GC-EAD, linalool was active on two flies only. An antennal response to the solvent peak of dichloromethane was noted in all GC-EAD runs. In EAG experiments, responses to a 10^−3^ dilution were compared to the response to paraffin oil (solvent) to determine which induced a larger antennal response. Four compounds can be highlighted: ethyl acetate [paired t-test, t_5_=-2.887, *p* = 0.034], isoamyl acetate [paired-t-test, t_5_= -2.708, *p* = 0.042], β-myrcene [paired t-test, t_5_= -2.195, *p* = 0.079], and linalool [paired t-test, t_5_= -2.368, *p* = 0.064], in addition to the 2-heptanone positive control [paired t-test, t_5_ = 4.174, *p* = 0.014]. Other GC-EAD- active compounds did not induce a response different from the response to paraffin oil in EAG experiments [paired t-test, *p* > 0.1]. Ethanol and the 11 other compounds identified from the two yeasts in CLSA-GC-MS were not antennally active in neither experiment. Lastly, males and females did not differ in their responses to any compound in EAG experiments [Wilcoxon sum-rank test, *p* > 0.05]. Responses to the positive control 2-heptanone did not differ between the start and end of experiments suggesting no antennal weakening.

## Discussion

The aim of the present study was to identify the most attractive yeasts to *D. suzukii* among the eight species tested, to characterize their volatile profiles and to assess which of these volatiles are detected by the fly via a comparative analysis of yeast headspace compositions. The chosen yeasts are naturally associated with *D. suzukii* (Hamby et al. 2012; Bellutti et al. 2018; Lewis et al. 2019; Jones et al. 2022) while *S. cerevisiae* was primarily selected for being a commonly used model in yeast research (Mortimer and Johnston 1986). The yeasts *S. vini* and *C. santaluciae* were only rarely reported from feeding galleries of *D. suzukii* larvae in grapes (Bellutti et al. 2018; Drumonde-Neves et al. 2020) and their attractiveness and headspace volatiles were here investigated in detail for the first time. Yeasts associated with *D. suzukii* represent an important resource for the development of control tools to facilitate monitoring, mass trapping or to improve the efficiency and specificity of insecticide application (Knight et al. 2016; Mori et al. 2017; Noble et al. 2019, 2021, 2022; Rehermann et al. 2022; Spitaler et al. 2022). Some attractive lures are already on the market for integration in pest management methods such as push-pull and attract-and-kill (Alkema et al. 2019; Klick et al. 2019; Rossi Stacconi et al. 2020; Urbaneja-Bernat et al. 2022). Notably, *H. uvarum* and other yeasts like *S. cerevisiae* and *I. terricola* tested in previous works, are efficient in luring flies into traps (Scheidler et al. 2015; Bueno et al. 2019; Kleman et al. 2022). All seven yeasts that are naturally associated with *D. suzukii* were more attractive than the reference strain of *S. cerevisiae* suggesting that yeasts represent specific habitat cues, which might contribute to the olfactory attraction to food sources in *D. suzukii* flies. *H. uvarum* 2.2, in particular stimulates feeding and promotes egg laying (Spitaler et al. 2020). The attractiveness of *H. uvarum*, which was one of the most attractive yeasts, is consistent with previous findings (Scheidler et al. 2015; Mori et al. 2017; Erdei et al. 2022). Furthermore, *H. uvarum* 2.2 was recently shown as a persistent attractive lure that can be applied on grape leaves as part of an attract-and-kill management method (Bianchi et al. 2020a; Rehermann et al. 2022; Spitaler et al. 2022). In our study we demonstrated that *S. vini* is more attractive than *H. uvarum* 2.2 in short range laboratory trapping assays, thus representing a novel candidate source to be developed into pest management control tools.

Three volatile collection techniques were used for a detailed and profound characterisation of yeast volatile profiles. Indeed, using different collection and analytical methods led to a larger number of compounds and better understanding of the qualitative and quantitative composition of yeast headspace samples. As we expected, several compounds were detected by the different collection techniques. Nevertheless, the techniques differed significantly in their outcomes, leading to distinct volatile profiles for the same yeasts. For instance, SPME permitted the detection of a large variety of chemicals released by the yeast cultures. This allowed to gain a deeper insight into the qualitative volatile profiles of the different yeast species, highlighting their unique fingerprint in VOC release. However, as the affinity with the SPME fibre varies per chemical, some compounds may be undetected, and the ratios and quantities of compounds released in the headspace may not be accurately reflected (Zhang and Pawliszyn 1996; García et al. 2019). The DHS method, which consist in a static collection, facilitated the identification of compound classes like short-chain acetates and alcohols which elute at a fast rate on the apolar column (LRI < 600) and compounds which were not picked up by SPME. Despite being emitted in high amounts, compounds like benzaldehyde were only detected in traces with the DHS technique. This can be attributed to the fact that the volatiles were collected from 1 mL of air only. The DHS method though, proved to be better for the detection of short-chain volatiles that were not visible with the CLSA method (ethyl acetate, propyl acetate, 2-methylpropyl acetate, ethanol, 2-methylpropan-1-ol, 3-methylbutan-1-ol; see Table 2), as it is for SPME but with the advantage of not being a passive method. The SPME method in fact involves an adsorbent material by passive diffusion, exposing the fibre to the vapour above the liquid. After a certain time, the analyte concentration in the SPME layer reaches an equilibrium level with its environment (García et al. 2019). With DHS, the proportion among the volatile components was kept naturally, as the volatile collection does not depend on the affinity of the compounds to absorbent matrices. The DHS-GC-MS method consists of a direct injection of headspace without volatile absorption and concentration, hereby preserving the most accurate representation of the real ratios of the volatile compounds in headspace. There were similarities in the array of volatiles produced by some of the species, but the concentration of these volatiles differed noticeably (Table 2). The CLSA coupled with air-entrainment is a dynamic headspace collection, which permitted to detect and quantify additional yeast-specific terpenes in the volatile profile of *S. vini*. This was particularly interesting given that it was the most attractive yeast. However, this technique required the use of a solvent to collect the volatiles, complicating the identification of compounds eluting before or simultaneously with the solvent. For instance, ethanol was not recorded by GC-MS because of its coelution with the solvent (Becher et al. 2012).

The composition of *S. vini* headspace is similar to the one described by Gamero et al. (2016) except for the release of several terpenoids, which were detected by three methods. Unlike all less attractive yeasts, *S. vini* released distinct terpenes among which, β-myrcene and linalool were antennally active. The terpenes from the GC eluent induced an antennal response in only a few male and female flies (25%) whereas esters (e.g., ethyl acetate) and ketones (2-heptanone) induced responses in 100% of the male and female flies tested. There is a natural variability among flies with compounds for which detection varies greatly, as shown by Keesey et al. (2015). Antennal responses variability was evident in the case of benzaldehyde, where data demonstrated a larger variation in the response (see Electronic Supplementary Table 2). Such variation most likely induced a non-significant response from the tested male and female antennas in the EAG tests (see Fig. 3d). All the male and female flies tested in the GC-EAD experiments responded to the positive control 2-heptanone as expected and demonstrated by previous studies (Dobritsa et al. 2003; Keesey et al. 2015; Lebreton et al. 2017). Males and females’ responses to volatiles did not differ confirming earlier work regarding a non-sex-specific detection of environmental volatiles in Drosophila (Shiao et al. 2013). β-Myrcene and linalool were found antennally active in earlier studies (Cloonan et al. 2018) and can therefore be considered candidate attractants in *S. vini* headspaces. In addition, *S. vini* released a significant amount of ethyl acetate, which was found attractive to *D. suzukii* (Feng et al. 2018). As reported in Shen et al. (2016), the yeast *S. vini* belongs to the family of Saccharomycopsidaceae and may have a distinct metabolic pathway from other yeasts studied. The data reported by Chen et al. (2018) showing the release of *de novo* synthesised terpenoid compounds by Saccharomycopsidaceae are confirmed by our results. Some of the *S. vini* terpenoids found in our study (linalool, citronellol, (*E*)-geraniol, α-terpineol) agree with those reported in Chen et al. (2018) and in addition, we detected β-myrcene, β-ocimene, (*Z*)-geraniol, geranial, (*E, Z*)-allocimene, (*E, E*)-allocimene, α-terpinene, γ-terpinene, cosmene, p-1,3,8-menthatriene, terpinolene, and β-phellandrene. The SPME analysis contributed massively to the identification of those terpenoids, present exclusively in the profile of *S. vini*. Interestingly, *S. vini* naturally inhabits grapes (Lachance et al. 2000) and may be part of the wine making process (König and Berkelmann-Löhnerz 2017). The emission of terpenes like myrcene, β-ocimene, linalool, citronellol, and (*Z*)-geraniol confer a peculiarity that differentiate this species from all the other analysed yeasts. It has been shown in previous research that *S. vini* can be a feeding stimulant and promotes the fecundity of *D. suzukii* females (Spitaler et al. 2020).

The second most attractive yeast to females, *H. uvarum* 2.2, unlike *S. vini*, released isoamyl acetate, which is attractive to *D. suzukii* (Revadi et al. 2015; Piñero et al. 2019). The attractivity of these two yeasts may therefore be mediated by the detection of two distinct headspace profiles. The three strains of *H. uvarum* were similar in attracting female flies and had a very similar volatile profile illustrating that yeast metabolites can display phylogenetic and ecological relations. Some of the volatiles characterized, notably ethyl acetate, isoamyl acetate and 2-phenylethanol were also detected earlier from different strains of *H. uvarum* (Scheidler et al. 2015; Erdei et al. 2022; Kleman et al. 2022). In a comparative study conducted by Piper et al. (2017) orange-agar solid medium was applied as substrate, which conferred a series of terpenoid volatiles to the yeasts profile that were not detected in our collection likely because of the use of different growth media.

Significant differences were found between the volatile profiles of the *H. uvarum* strains and *S. cerevisiae*, systematizing into two genetically close families (Saccharomycodaceae and Saccharomycetaceae respectively) as reported in Shen et al. (2016). The yeast *S. cerevisiae* was previously investigated with respect to its interaction with drosophilid flies though volatile compounds. Arguello et al. (2013) and Becher et al. (2018) reported ethanol, 3-methyl butanol, and 2-phenylethanol as three of the most abundant alcohols present in *S. cerevisiae* volatile profile, which is in line with our findings. Bueno et al. (2019) also reported a few differences among the volatile profiles of microbial strains, linking the variation in attractiveness to the volatile profile of the studied microorganism. The emission of ethanol for example, was significantly higher in *S. cerevisiae* and *M. pulcherrima* compared to other yeasts in our study. These two yeasts have previously been reported as feeding deterrents of *D. suzukii* larvae (Spitaler et al. 2020). The yeast *M. pulcherrima* belongs to the family of Metschnikowiaceae like *C. santaluciae* (Shen et al. 2016; Drumonde-Neves et al. 2020). In addition, previous findings on *M. pulcherrima* report that its volatile composition features a fair number of alcohols (Ljunggren et al. 2019). While we found that *S. cerevisiae* and *M. pulcherrima* shared ethyl acetate and most of the alcohols, *C. santaluciae* had a higher number of acetates in its profile, with ethyl acetate quantitatively standing out above all other yeasts in DHS-GC-MS. The yeast *I. terricola*, belonging to the family of Pichiaceae, which is phylogenetically close to Metschnikowiaceae (Shen et al. 2016), released esters, unlike *M. pulcherrima*. Surprisingly, the volatile profile of *I. terricola* was more similar to *S. cerevisiae* compared to other yeasts, despite their families (Pichiaceae and Saccharomycetaceae, respectively) being phylogenetically distant (Shen et al. 2016). Overlap in the emission of volatiles had been shown for phylogenetically distantly related yeasts before, and *S. cerevisiae* shares several orthologous genes involved in the biosynthetic pathways of VOCs with yeasts that are phylogenetically and ecologically distinct (Becher et al. 2018).

We also assessed whether ethanol was detected as *S. cerevisiae*, the least attractive to females in our behavioural study, released the highest amount of ethanol, while more attractive yeasts released the lowest amounts. Indeed, *S. cerevisiae* and its close relatives are able to out-compete other microorganisms by the accumulation of metabolites (Piškur and Langkjaer 2004), however ethanol did not seem to induce the lack of cultivability of *H. uvarum* in presence of *S. cerevisiae* (Wang et al. 2015). In *D. suzukii* the tolerance towards ethanol is low (Chakraborty et al. 2022). The association with *H. uvarum* as a comparatively poor producer of ethanol might protect the fly larvae from high concentrations of toxic alcohol, which might have contributed to a mutualistic relation between *D. suzukii* and *H. uvarum* (Chakraborty et al. 2022). Interestingly, our analyses of DHS showed similarly low production of ethanol in *H. uvarum* and *S. vini* as a shared feature between the most attractive yeasts. However, our assays could not reveal that ethanol is antennally active in the conditions tested (dilutions from 10^−2^ to 10^−8^ in paraffin oil in EAG, and neat headspace in GC-EAD). Antennal activity for ethanol has previously been reported for *D. suzukii*, but with low consistency between replicated antennal recordings (Cha et al. 2012). Ethanol has previously been found as relevant component in the formulation of attractive lures and is applied in traps targeting *D. suzukii* (Cha et al. 2012, 2014; Feng et al. 2018). Still, the detection mechanism together with a chemosensory receptor remain to be deciphered.

The only volatile commonly detected by *D. suzukii* males and females across all the eight studied yeasts with DHS-GC-MS was 2-phenylethanol, which appears as an ancient and evolutionary conserved compound in yeasts, as well as ancient angiosperms (Becher et al. 2018). The yeasts *M. pulcherrima*, *I. terricola*, and *S. cerevisiae* showed the highest amount of 2-phenylethanol emission. It is mentioned in previous studies that some volatiles, such as 2-phenylethanol, are produced *de novo* by both plants and yeasts, via different pathways (Williams et al. 1983; Chantasuban et al. 2018; Chreptowicz et al. 2018). Other yeast volatiles, like ethyl hexanoate, nonanal, decanal, 2-phenylethyl acetate, are also commonly known from flowers and fruit volatiles, and yeasts that are associated with flowers may contribute to floral scent (Tieman et al. 2007; Stökl et al. 2010; Becher et al. 2018). Moreover, *I. terricola* and *S. cerevisiae* were distinct in the production of esters.

To conclude, in our research we identified *S. vini* and *H. uvarum* as attractive yeasts that should be further investigated for possible implementation in monitoring and attract-and-kill methods within integrated pest management (IPM) of *D. suzukii*. A further option could be to test their combination for increased attractiveness compared to the individual yeasts. We described and compared volatile profiles of *Drosophila*-associated yeasts and discovered species-specific and *H. uvarum* strain-specific volatile profiles. The headspace volatiles of *S. vini* and *C. santaluciae* were investigated in detail for the first time using diverse sampling techniques. Lastly, we highlighted the *S. vini* terpenes β-myrcene, linalool and the ester ethyl acetate for their electrophysiological activity in *D. suzukii*. The studied yeasts and volatiles represent important resources that might improve the efficiency and specificity of management methods targeting *D. suzukii*.

## Supplementary Information

Below is the link to the electronic supplementary material.

ESM 1 (XLSX 18.6 KB)

ESM 2 (XLSX 24.3 KB)

ESM 3 Total percentage of flies trapped in liquid yeast cultures, the culture medium Potato Dextrose Broth (PDB) or not making a choice (NC) after 24 h. The bar plots show the cumulative results of six replicates (with 20 flies each) per treatment. For each yeast, different letters mean significant differences between trappings of specific yeast, PDB and NC. (GLM-Tukey contrast; *p* < 0.05). *H.u., Hanseniaspora uvarum; S.c., Saccharomyces cerevisiae; I.t., Issatchenkia terricola; C.sa., Clavispora santaluciae; S.v., Saccharomycopsis vini; M.p., Metschnikowia pulcherrima*. (JPEG 1.39 MB)

## Data Availability

No datasets were generated or analysed during the current study.

## References

[CR1] Abraham J, Zhang A, Angeli S, Abubeker S, Michel C, Feng Y, Rodriguez-Saona C (2015) Behavioral and antennal responses of Drosophila suzukii (Diptera: Drosophilidae) to volatiles from fruit extracts. Environ Entomol 44:356–367. 10.1093/ee/nvv013 26313190 10.1093/ee/nvv013

[CR2] Abraham J, Angeli S, Antwi JB, Rodriguez-Saona C (2022) Research advances on Drosophila suzukii. Front Ecol Evol 10:897222. 10.3389/fevo.2022.897222

[CR3] Alkema JT, Dicke M, Wertheim B (2019) Context-dependence and the development of push-pull approaches for integrated management of *Drosophila suzukii*. Insects 10:454. 10.3390/insects10120454 31847450 10.3390/insects10120454 PMC6956413

[CR4] Arguello JR, Sellanes C, Lou YR, Raguso RA (2013) Can yeast (*S. Cerevisiae*) metabolic volatiles provide polymorphic signaling? PLoS ONE 8(8):e70219. 10.1371/journal.pone.0070219 23990899 10.1371/journal.pone.0070219 PMC3747187

[CR5] Atallah J, Teixeira L, Salazar R, Zaragoza G, Kopp A (2014) The making of a pest: the evolution of a fruit-penetrating ovipositor in *Drosophila suzukii* and related species. Proc R Soc B Biol Sci 281:20132840. 10.1098/rspb.2013.2840 10.1098/rspb.2013.2840 PMC3953835 24573846

[CR6] Bates D, Mächler M, Bolker B, Walker S (2015) Fitting linear mixed-effects models using lme4. J Stat Softw 67(1):1–48. 10.18637/jss.v067.i01

[CR7] Becher PG, Flick G, Rozpędowska E, Schmidt A, Hagman A, Lebreton S, Larsson MC, Hansson BS, Piškur J, Witzgall P, Bengtsson M, Thompson K (2012) Yeast, not fruit volatiles mediate *Drosophila melanogaster* attraction, oviposition and development. Funct Ecol 26:822–828. 10.1111/j.1365-2435.2012.02006.x

[CR8] Becher PG, Hagman A, Verschut V, Chakraborty A, Rozpędowska E, Lebreton S, Bengtsson M, Flick G, Witzgall P, Piškur J (2018) Chemical signaling and insect attraction is a conserved trait in yeasts. Ecol Evol 8:2962–2974. 10.1002/ece3.3905 29531709 10.1002/ece3.3905 PMC5838033

[CR9] Bellutti N, Gallmetzer A, Innerebner G, Schmidt S, Zelger R, Koschier EH (2018) Dietary yeast affects preference and performance in *Drosophila suzukii*. J Pest Sci 91:651–660. 10.1007/s10340-017-0932-2 10.1007/s10340-017-0932-2 PMC5847167 29568250

[CR10] Benito N, Lopes-da-Silva M, Sivori Silva dos Santos R (2016) Potential spread and economic impact of invasive *Drosophila suzukii* in Brazil. Pesq Agropec Bras 51:571–578. 10.1590/S0100-204X2016000500018

[CR11] Bianchi F, Spitaler U, Castellan I, Cossu CS, Brigadoi T, Duménil C, Angeli S, Robatscher P, Vogel RF, Schmidt S, Eisenstecken D (2020a) Persistence of a yeast-based (Hanseniaspora uvarum) attract-and-kill formulation against Drosophila suzukii on grape leaves. Insects 11:810. 10.3390/insects11110810 33217960 10.3390/insects11110810 PMC7698740

[CR12] Bianchi F, Spitaler U, Robatscher P, Vogel RF, Schmidt S, Eisenstecken D (2020b) Comparative lipidomics of different yeast species associated to Drosophila suzukii. Metabolites 10(9):352. 10.3390/metabo10090352 32872268 10.3390/metabo10090352 PMC7569767

[CR13] Blouquy L, Mottet C, Olivares J, Plantamp C, Siegwart M, Barrès B (2021) How varying parameters impact insecticide resistance bioassay: an example on the worldwide invasive pest Drosophila suzukii. PLoS ONE 16:e0247756. 10.1371/journal.pone.0247756 33667239 10.1371/journal.pone.0247756 PMC7935283

[CR14] Bueno E, Martin KR, Raguso RA, Mcmullen JG II, Hesler SP, Loeb GM, Douglas AE (2019) Response of wild spotted wing drosophila (Drosophila suzukii) to microbial volatiles. J Chem Ecol 46(8):688–698. 10.1007/s10886-019-01139-4 31879864 10.1007/s10886-019-01139-4

[CR15] Cai P, Song Y, Yi C, Zhang Q, Xia H, Lin J, Zhang H, Yang J, Ji Q, Chen J (2019) Potential host fruits for Drosophila suzukii: olfactory and oviposition preferences and suitability for development. Entomol Exp Appl 167:880–890. 10.1111/eea.12840

[CR16] Cha DH, Adams T, Rogg H, Landolt PJ (2012) Identification and field evaluation of fermentation volatiles from wine and vinegar that mediate attraction of spotted wing Drosophila, Drosophila suzukii. J Chem Ecol 38:1419–1431. 10.1007/s10886-012-0196-5 23065086 10.1007/s10886-012-0196-5

[CR17] Cha DH, Adams T, Christopher TW, Sampson BJ, Adamczyk JJ Jr, Rogg H, Landolt PH (2014) A four-component synthetic attractant for *Drosophila suzukii* (Diptera: Drosophilidae) isolated from fermented bait headspace. Pest Manag Sci 70:324–331. 10.1002/ps.3568 23633121 10.1002/ps.3568

[CR18] Chakraborty A, Mori B, Rehermann G, Hernández Garcia A, Lemmen-Lechelt J, Hagman A, Khalil S, Håkansson S, Witzgall P, Becher PG (2022) Yeast and fruit fly mutual niche construction and antagonism against mould. Funct Ecol 36:1639–1654. 10.1111/1365-2435.14054

[CR19] Chantasuban T, Santomauro F, Gore-Lloyd D, Parsons S, Henk D, Scott RJ, Chuck C (2018) Elevated production of the aromatic fragrance molecule, 2-phenylethanol, using Metschnikowia pulcherrima through both de novo and ex novo conversion in batch and continuous modes. J Chem Technol Biotechnol 93(8):2118–2130. 10.1002/jctb.5597 30069076 10.1002/jctb.5597 PMC6055805

[CR20] Chen WT, Bai M, Tang WY, Tan J, Wang MJ, Yang GS (2018) A Saccharomycopsis vini strain capable of synthesizing monoterpenes de novo. Mycosystema 37(6):703–711. 10.13346/j.mycosystema.180051. (in Chinese)

[CR21] Chong J, Soufan O, Li C, Caraus I, Li S, Bourque G, Wishart DS, Xia J (2018) MetaboAnalyst 4.0: towards more transparent and integrative metabolomics analysis. Nucleic Acids Res 46:486–494. 10.1093/nar/gky310 10.1093/nar/gky310 PMC6030889 29762782

[CR22] Chreptowicz K, Sternicka MK, Kowalska PD, Mierzejewska J (2018) Screening of yeasts for the production of 2-phenylethanol (rose aroma) in organic waste-based media. Lett Appl Microbiol 66(2):153–160. 10.1111/lam.12835 29224193 10.1111/lam.12835

[CR23] Cini A, Ioriatti C, Anfora G (2012) A review of the invasion of Drosophila suzukii in Europe and a draft research agenda for integrated pest management. Bull Insectol 65:149–160 (http://hdl.handle.net/10449/21029)

[CR24] Cloonan KR, Abraham J, Angeli S, Syed Z, Rodriguez-Saona C (2018) Advances in the chemical ecology of the spotted wing drosophila (*Drosophila suzukii*) and its applications. J Chem Ecol 44(10):922–939. 10.1007/s10886-018-1000-y 30054769 10.1007/s10886-018-1000-y

[CR25] Crava CM, Romani R, Zanini D, Amati S, Sollai G, Crnjar R, Haase A, Paoli M, Rossi-Stacconi MV, Rota-Stabelli O, Tait G, Anfora G (2020) Structural and transcriptional evidence of mechanotransduction in the Drosophila suzukii ovipositor. J Insect Physiol 125:104088. 10.1016/j.jinsphys.2020.104088 32652080 10.1016/j.jinsphys.2020.104088

[CR26] Dobritsa AA, van der Goes van Naters W, Warr CG, Steinbrecht RA, Carlson JR (2003) Integrating the molecular and cellular basis of odor coding in the Drosophila antenna. Neuron 37(5):827–841. 10.1016/S0896-6273(03)00094-1 10.1016/s0896-6273(03)00094-1 12628173

[CR27] Drumonde-Neves J, Čadež N, Reyes-Domínguez Y, Gallmetzer A, Schuller D, Lima T, Pais C, Franco-Duarte R (2020) Clavispora santaluciae f.a., sp. nov., a novel ascomycetous yeast species isolated from grapes. Int J Syst Evol Microbiol 70(12):6307–6312. 10.1099/ijsem.0.004531 33090949 10.1099/ijsem.0.004531

[CR28] Ðurović G, Maddalena G, Alawamleh A, Guzzon R, Mazzoni V, Ioriatti C, Dalton D, Walton VM, Suckling DM, Butler RC, Angeli S, De Cristofaro A, Anfora G (2020) Liquid baits with Oenococcus oeni increase captures of Drosophila suzukii. Insects 12:66. 10.3390/insects12010066 10.3390/insects12010066 PMC7828427 33450937

[CR29] Erdei AL, Szelényi MO, Deutsch F, Rikk P, Molnár BP (2022) Lure design for Drosophila suzukii based on liquid culture of fruit epiphytic yeasts: comparing the attractivity of fermentation volatiles for seasonal morphs. J Appl Entomol 146:773–785. 10.1111/jen.13006

[CR30] Farnsworth D, Hamby K, Bolda M, Goodhue R, Williams J, Zalom F (2017) Economic analysis of revenue losses and control costs associated with the spotted wing drosophila, *Drosophila suzukii* (Matsumura), in the California raspberry industry. Pest Manag Sci 73:1083–1090. 10.1002/ps.4497 27943618 10.1002/ps.4497

[CR31] Del Fava E, Ioriatti C, Melegaro A (2017) Cost-benefit analysis of controlling the spotted wing drosophila (Drosophila suzukii (Matsumura)) spread and infestation of soft fruits in Trentino, Northern Italy. Pest Manag Sci 73:2318–2327. 10.1002/ps.4618 28523823 10.1002/ps.4618

[CR32] Feng Y, Bruton R, Park A, Zhang A (2018) Identification of attractive blend for spotted wing drosophila, Drosophila suzukii, from apple juice. J Pest Sci 91:1251–1267. 10.1007/s10340-018-1006-9 10.1007/s10340-018-1006-9 PMC6063330 30100831

[CR33] Gamero A, Quintilla R, Groenewald M, Alkema W, Boekhout T, Hazelwood L (2016) High-throughput screening of a large collection of non-conventional yeasts reveals their potential for aroma formation in food fermentation. Food Microbiol 60:147–159. 10.1016/j.fm.2016.07.006 27554157 10.1016/j.fm.2016.07.006

[CR34] García YM, Rufini JCM, Campos MP, Guedes MNS, Augusti R, Melo JOF (2019) SPME fiber evaluation for volatile organic compounds extraction from acerola. J Braz Chem Soc 30(2):247–255. 10.21577/0103-5053.20180173

[CR35] Gress B, Zalom F (2019) Identification and risk assessment of spinosad resistance in a California population of *Drosophila suzukii*. Pest Manag Sci 75:1270–1276. 10.1002/ps.5240 30324771 10.1002/ps.5240

[CR36] Hamby KA, Becher PG (2016) Current knowledge of interactions between *Drosophila suzukii* and microbes, and their potential utility for pest management. J Pest Sci 89:621–630. 10.1007/s10340-016-0768-1

[CR37] Hamby KA, Hernández A, Boundy-Mills K, Zalom FG (2012) Associations of yeasts with spotted-wing Drosophila (*Drosophila suzukii*; Diptera: Drosophilidae) in cherries and raspberries. Appl Environ Microbiol 78:4869–4873. 10.1128/AEM.00841-12 22582060 10.1128/AEM.00841-12 PMC3416361

[CR38] Hauser M (2011) A historic account of the invasion of Drosophila suzukii (Matsumura) (Diptera: Drosophilidae) in the continental United States, with remarks on their identification. Pest Manag Sci 67:1352–1357. 10.1002/ps.2265 21898759 10.1002/ps.2265

[CR39] Haye T, Girod P, Cuthbertson AGS, Wang XG, Daane KM, Hoelmer KA, Baroffio C, Zhang JP, Desneux N (2016) Current SWD IPM tactics and their practical implementation in fruit crops across different regions around the world. J Pest Sci 89:643–651. 10.1007/s10340-016-0737-8

[CR40] Hothorn T, Bretz F, Westfall P (2008) Simultaneous inference in general parametric models. Biom J 50(3):346–363. 10.1002/bimj.200810425 18481363 10.1002/bimj.200810425

[CR41] Iglesias LE, Nyoike TW, Liburd OE (2014) Effect of trap design, bait type, and age on captures of *Drosophila suzukii* (Diptera: Drosophilidae) in berry crops. J Econ Entomol 107:1508–1518. 10.1603/EC13538 25195443 10.1603/ec13538

[CR42] Ioriatti C, Walton V, Dalton D, Anfora G, Grassi A, Maistri S, Mazzoni V (2015) *Drosophila suzukii* (Diptera: Drosophilidae) and its potential impact to wine grapes during harvest in two cool climate wine grape production regions. J Econ Entomol 108(3):1148–1155. 10.1093/jee/tov042 26470240 10.1093/jee/tov042

[CR43] Jones R, Fountain MT, Andreani NA, Günther CS, Goddard MR (2022) The relative abundances of yeasts attractive to *Drosophila suzukii* differ between fruit types and are greatest on raspberries. Sci Rep 12(1):10382. 10.1038/s41598-022-14275-x 35725889 10.1038/s41598-022-14275-x PMC9209449

[CR44] Karageorgi M, Bräcker L, Lebreton S, Minervino C, Cavey M, Siju K, Kadow I, Gompel N, Prud’homme B (2017) Evolution of multiple sensory systems drives novel egg-laying behavior in the fruit pest *Drosophila suzukii*. Curr Biol 27:847–853. 10.1016/j.cub.2017.01.055 28285999 10.1016/j.cub.2017.01.055 PMC5364372

[CR45] Keesey IW, Knaden M, Hansson BS (2015) Olfactory specialization in Drosophila suzukii supports an ecological shift in host preference from rotten to fresh fruit. J Chem Ecol 41:121–128. 10.1007/s10886-015-0544-3 25618323 10.1007/s10886-015-0544-3 PMC4351439

[CR46] Kienzle R, Groß LB, Caughman S, Rohlfs M (2020) Resource use by individual Drosophila suzukii reveals a flexible preference for oviposition into healthy fruits. Sci Rep 10:3132. 10.1038/s41598-020-59595-y 32081929 10.1038/s41598-020-59595-y PMC7035383

[CR47] Kim H, Kim Y, Roh GH, Kim YH (2023) Comparison of preference for chemicals associated with fruit fermentation between Drosophila melanogaster and Drosophila suzukii and between virgin and mated D. Melanogaster. Insects 14:382. 10.3390/insects14040382 37103197 10.3390/insects14040382 PMC10145260

[CR48] Kleman I, Rehermann G, Kwadha CA, Witzgall P, Becher PG (2022) *Hanseniaspora uvarum* attracts *Drosophila suzukii* (Diptera: Drosophilidae) with high specificity. J Econ Entomol 115(4):999–1007. 10.1093/jee/toac029 35385117 10.1093/jee/toac029 PMC9365507

[CR49] Klick J, Yang W, Walton V, Dalton D, Hagler J, Dreves A, Lee J, Bruck D (2016) Distribution and activity of Drosophila suzukii in cultivated raspberry and surrounding vegetation. J Appl Entomol 140:37–46. 10.1111/jen.12234

[CR50] Klick J, Rodriguez-Saona CR, Cumplido JH et al (2019) Testing a novel attract-and-kill strategy for *Drosophila suzukii* (Diptera: Drosophilidae). J Insect Sci 19(1):3. 10.1093/jisesa/iey132 30624704 10.1093/jisesa/iey132 PMC6324652

[CR51] Knapp L, Mazzi D, Finger R (2021) The economic impact of Drosophila suzukii: perceived costs and revenue losses of Swiss cherry, plum and grape growers. Pest Manag Sci 77:978–1000. 10.1002/ps.6110 32990345 10.1002/ps.6110 PMC7821377

[CR52] Knight AL, Basoalto E, Yee W, Hilton R, Kurtzman CP (2016) Adding yeasts with sugar to increase the number of effective insecticide classes to manage *Drosophila suzukii* (Matsumura) (Diptera: Drosophilidae) in cherry. Pest Manag Sci 72:1482–1490. 10.1002/ps.4171 26454150 10.1002/ps.4171

[CR53] Kolde R (2019) Pheatmap: pretty heatmaps. R package version 1.0.12. https://CRAN.R-project.org/package=pheatmap. Accessed 1 Apr 2024

[CR54] König H, Berkelmann-Löhnerz B (2017) Maintenance of wine-associated microorganisms. In: König H, Unden G, Fröhlich J (eds) Biology of microorganisms on grapes, in must and in wine. Springer, Berlin Heidelberg, pp 554–556

[CR55] Kwadha CA, Okwaro LA, Kleman I, Rehermann G, Revadi S, Ndlela S, Khamis FM, Nderitu PW, Kasina M, George MK, Kithusi GG, Mohamed SA, Lattorff HMG, Becher PG (2021) Detection of the spotted wing drosophila, *Drosophila suzukii*, in continental Sub-saharan Africa. J Pest Sci 94:251–259. 10.1007/s10340-021-01330-1

[CR56] Lachance MA, Pupovac-Velikonja A, Natarajan S, Schlag-Edler B (2000) Nutrition and phylogeny of predacious yeasts. Can J Microbiol 46(6):495–505. 10.1139/w00-021 10913970 10.1139/w00-021

[CR57] Landolt PJ, Adams T, Davis TS, Rogg H (2012a) Spotted wing drosophila, Drosophila suzukii (Diptera: Drosophilidae), trapped with combinations of wines and vinegars. Fla Entomol 95:326–332. 10.1653/024.095.0213

[CR58] Landolt PJ, Adams T, Rogg H (2012b) Trapping spotted wing drosophila, Drosophila suzukii (Matsumura) (Diptera: Drosophilidae), with combinations of vinegar and wine, and acetic acid and ethanol. J Appl Entomol 136:148–154. 10.1111/j.1439-0418.2011.01646.x

[CR59] Lasa R, Navarro-de-la-Fuente L, Gschaedler-Mathis AC, Kirchmayr MR, Williams T (2019) Yeast species, strains, and growth media mediate attraction of Drosophila suzukii (Diptera: Drosophilidae). Insects 10:228. 10.3390/insects10080228 31370207 10.3390/insects10080228 PMC6722520

[CR60] Lebreton S, Borrero-Echeverry F, Gonzalez F, Solum M, Wallin EA, Hedenström E et al (2017) A Drosophila female pheromone elicits species-specific long-range attraction via an olfactory channel with dual specificity for sex and food. BMC Biol 15:88. 10.1186/s12915-017-0427-x 28962619 10.1186/s12915-017-0427-x PMC5622430

[CR61] Lee JC, Bruck DJ, Curry H, Edwards D, Haviland DR, van Steenwyk RA, Yorgey BM (2011) The susceptibility of small fruits and cherries to the spotted-wing drosophila, Drosophila suzukii. Pest Manag Sci 67:1358–1367. 10.1002/ps.2225 21710685 10.1002/ps.2225

[CR62] Lee JC, Dalton DT, Swoboda-Bhattarai KA, Bruck DJ, Burrack HJ, Strik BC, Woltz JM, Walton VM (2016) Characterization and manipulation of fruit susceptibility to Drosophila suzukii. J Pest Sci 89:771–780. 10.1007/s10340-015-0692-9

[CR63] Lewis MT, Koivunen EE, Swett CL, Hamby KA (2019) Associations between Drosophila suzukii (Diptera: Drosophilidae) and fungi in raspberries. Environ Entomol 48:68–79. 10.1093/ee/nvy167 30520973 10.1093/ee/nvy167

[CR64] Ljunggren J, Borrero-Echeverry F, Chakraborty A, Lindblom TUT, Hedenström E, Karlsson M, Witzgall P, Bengtsson M (2019) Yeast volatomes differentially affect larval feeding in an insect herbivore. Appl Environ Microbiol 85(21):e01761–e01719. 10.1128/AEM.01761-19 31444202 10.1128/AEM.01761-19 PMC6803314

[CR65] Marchand PA (2023) Evolution of plant protection active substances in Europe: the disappearance of chemicals in favour of biocontrol agents. Environ Sci Pollut Res Int 30:1–17. 10.1007/s11356-022-24057-7 36378372 10.1007/s11356-022-24057-7

[CR66] Matheu MP, Cahalan MD, Parker I (2011) General approach to adoptive transfer and cell labeling for immunoimaging. Cold Spring Harb Protoc 1(2):pdb.prot5565. 10.1101/pdb.prot5565 10.1101/pdb.prot5565 21285265

[CR67] Mazzi D, Bravin E, Meraner M, Finger R, Kuske S (2017) Economic impact of the introduction and establishment of Drosophila suzukii on sweet cherry production in Switzerland. Insects 8:18. 10.3390/insects8010018 28208692 10.3390/insects8010018 PMC5371946

[CR68] Mori BA, Whitener AB, Leinweber Y, Revadi S, Beers EH, Witzgall P, Becher PG (2017) Enhanced yeast feeding following mating facilitates control of the invasive fruit pest Drosophila suzukii. J Appl Ecol 54:170–177. 10.1111/1365-2664.12688

[CR69] Mortimer RK, Johnston JR (1986) Genealogy of principal strains of the yeast genetic stock center. Genetics 113(1):35–43. 10.1093/genetics/113.1.35 3519363 10.1093/genetics/113.1.35 PMC1202798

[CR70] Noble R, Dobrovin-Pennington A, Phillips A, Cannon MF, Shaw B, Fountain MT (2019) Improved insecticidal control of spotted wing drosophila (Drosophila suzukii) using yeast and fermented strawberry juice baits. Crop Prot 125:104902. 10.1016/j.cropro.2019.104902

[CR71] Noble R, Walker A, Whitfield C, Harris A, Dobrovin-Pennington A, Fountain MT (2021) Minimizing insecticides for control of spotted wing drosophila (Drosophila suzukii) in soft fruit using bait sprays. J Appl Entomol 145:1–9. 10.1111/jen.12917

[CR72] Noble R, Shaw B, Walker A, Whitfield CE, Deaking G, Harris A, Dobrovin–Pennington A, Fountain MT (2022) Control of spotted wing drosophila (Drosophila suzukii) in sweet cherry and raspberry using bait sprays. J Pest Sci. 10.1007/s10340-022-01566-5 10.1007/s10340-025-01925-y PMC12546283 41141313

[CR73] Ometto L, Cestaro A, Ramasamy S, Grassi A, Revadi S, Siozios S, Moretto M, Fontana P, Varotto C, Pisani D, Dekker T, Wrobel N, Viola R, Pertot I, Cavalieri D, Blaxter M, Anfora G, Rota-Stabelli O (2013) Linking genomics and ecology to investigate the complex evolution of an invasive Drosophila pest. Genome Biol Evol 5(4):745–757. 10.1093/gbe/evt034 23501831 10.1093/gbe/evt034 PMC3641628

[CR74] Piñero JC, Barrett BA, Grant Bolton L, Follett PA (2019) β-cyclocitral synergizes the response of adult *Drosophila suzukii* (Diptera: Drosophilidae) to fruit juices and isoamyl acetate in a sex-dependent manner. Sci Rep 9:10574. 10.1038/s41598-019-47081-z 31332263 10.1038/s41598-019-47081-z PMC6646655

[CR75] Piper AM, Farnier K, Linder T, Speight R, Cunningham JP (2017) Two gut-associated yeasts in a tephritid fruit fly have contrasting effects on adult attraction and larval survival. J Chem Ecol 43:891–901. 10.1007/s10886-017-0877-1 28836040 10.1007/s10886-017-0877-1

[CR76] Piškur J, Langkjaer RB (2004) Yeast genome sequencing: the power of comparative genomics. Mol Microbiol 53:381–389. 10.1111/j.1365-2958.2004.04182.x 15228521 10.1111/j.1365-2958.2004.04182.x

[CR77] R Core Team (2020) R: a language and environment for statistical computing. R Foundation for Statistical Computing, Vienna

[CR78] Rehermann G, Spitaler U, Sahle K, Cossu CS, Delle Donne L, Bianchi F, Eisenstecken D, Angeli S, Schmidt S, Becher PG (2022) Behavioral manipulation of *Drosophila suzukii* for pest control: high attraction to yeast enhances insecticide efficacy when applied on leaves. Pest Manag Sci 78:896–904. 10.1002/ps.6699 34716651 10.1002/ps.6699

[CR79] Revadi S, Vitagliano S, Rossi-Stacconi M, Ramasamy S, Mansourian S, Carlin S, Vrhovsek U, Becher PG, Mazzoni V, Rota-Stabelli O (2015) Olfactory responses of *Drosophila suzukii* females to host plant volatiles. Physiol Entomol 40:54–64. 10.1111/phen.12088

[CR80] Rombaut A, Guilhot R, Xuéreb A, Benoit L, Chapuis M, Pierre-Gibert P, Fellous S (2017) Invasive *Drosophila suzukii* facilitates *Drosophila melanogaster* infestation and sour rot outbreaks in vineyards. Royal Soc Open Sci 4:170117. 10.1098/rsos.170117 10.1098/rsos.170117 PMC5383864 28405407

[CR81] Rossi Stacconi MV, Tait G, Rendon D, Grassi A, Boyer G, Nieri R, Walton VM (2020) Gumming up the works: field tests of a new food-grade gum as behavioral disruptor for *Drosophila suzukii* (Diptera: Drosophilidae). J Econ Entomol 113(4):1872–1880. 10.1093/jee/toaa072 32333602 10.1093/jee/toaa072

[CR82] Ruebenbauer A, Schlyter F, Hansson BS, Lofstedt C, Larsson MC (2008) Genetic variability and robustness of host odor preference in *Drosophila melanogaster*. Curr Biol 18:1438–1443. 10.1016/j.cub.2008.08.062 18804372 10.1016/j.cub.2008.08.062

[CR83] Scheidler NH, Liu Cheng, Hamby KA, Frank Zalom FG, Syed Z (2015) Volatile codes: correlation of olfactory signals and reception in Drosophila-yeast chemical communication. Sci Rep 5:14059. 10.1038/srep14059 26391997 10.1038/srep14059 PMC4585764

[CR84] Schoug A, Olsson J, Carlfors J, Schnürer J, Håkansson S (2006) Freeze-drying of *Lactobacillus coryniformis* Si3-effects of sucrose concentration, cell density, and freezing rate on cell survival and thermophysical properties. Cryobiology 53(1):119–127. 10.1016/j.cryobiol.2006.04.003 16756971 10.1016/j.cryobiol.2006.04.003

[CR85] Shawer R, Tonina L, Tirello P, Duso C, Mori N (2018) Laboratory and field trials to identify effective chemical control strategies for integrated management of *Drosophila suzukii* in European cherry orchards. Crop Prot 103:73–80. 10.1016/j.cropro.2017.09.010

[CR86] Shen XX, Zhou X, Kominek J, Kurtzman CP, Hittinger CT, Rokas A (2016) Reconstructing the backbone of the Saccharomycotina yeast phylogeny using genome-scale data. G3: genes, genomes. Genetics 6(12):3927–3939. 10.1534/g3.116.034744 10.1534/g3.116.034744 PMC5144963 27672114

[CR87] Shiao MS, Fan WL, Fang S et al (2013) Transcriptional profiling of adult Drosophila antennae by high-throughput sequencing. Zool Stud 52:42. 10.1186/1810-522X-52-42

[CR88] Spitaler U, Bianchi F, Eisenstecken D, Castellan I, Angeli S, Dordevic N, Robatscher P, Vogel RF, Koschier EH, Schmidt S (2020) Yeast species affects feeding and fitness of Drosophila suzukii adults. J Pest Sci 93:1295–1309. 10.1007/s10340-020-01266-y

[CR89] Spitaler U, Cossu CS, Delle Donne L, Bianchi F, Rehermann G, Eisenstecken D, Castellan I, Dumenil C, Angeli S, Robatscher P, Becher PG, Koschier EH, Schmidt S (2022) Field and greenhouse application of an attract-and-kill formulation based on the yeast *Hanseniaspora uvarum* and the insecticide spinosad to control *Drosophila suzukii* in grapes. Pest Manag Sci 78:1287–1295. 10.1002/ps.6748 34854220 10.1002/ps.6748 PMC9299924

[CR90] Stökl J, Strutz A, Dafni A, Svatos A, Doubsky J, Knaden M, Sachse S, Hansson BS, Stensmyr MC (2010) A deceptive pollination system targeting drosophilids through olfactory mimicry of yeast. Curr Biol 20(20):1846–1852. 10.1016/j.cub.2010.09.033 20933425 10.1016/j.cub.2010.09.033

[CR91] Tait G, Mermer S, Stockton D, Lee J, Avosani S, Abrieux A, Anfora G, Beers E, Biondi A, Burrack H et al (2021) *Drosophila suzukii* (Diptera: Drosophilidae): a decade of research towards a sustainable integrated pest management program. J Econ Entomol 114:1950–1974. 10.1093/jee/toab158 34516634 10.1093/jee/toab158

[CR92] Tieman DM, Loucas HM, Kim JY, Clark DG, Klee HJ (2007) Tomato phenylacetaldehyde reductases catalyze the last step in the synthesis of the aroma volatile 2-phenylethanol. Phytochemistry 68(21):2660–2669. 10.1016/j.phytochem.2007.06.005 17644147 10.1016/j.phytochem.2007.06.005

[CR93] Urbaneja-Bernat P, Cloonan K, Zhang A, Salazar-Mendoza P, Rodriguez-Saona C (2021) Fruit volatiles mediate differential attraction of Drosophila suzukii to wild and cultivated blueberries. J Pest Sci 94:1249–1263. 10.1007/s10340-021-01332-z

[CR94] Urbaneja-Bernat P, Holdcraft R, Hernández‐Cumplido J, Rhodes EM, Liburd OE, Sial AA, Mafra‐Neto A, Rodriguez‐Saona C (2022) Field, semi‐field and greenhouse testing of HOOK SWD, a SPLAT‐based attract‐and‐kill formulation to manage spotted‐wing drosophila. J Appl Entomol 146:1230–1242. 10.1111/jen.13073

[CR95] Venables WN, Ripley BD (2003) Modern applied statistics with S, 4th edn. Springer, New York. http://www.stats.ox.ac.uk/pub/MASS4/. ISBN 0-387-95457-0. Accessed 1 Apr 2024

[CR96] Walsh DB, Bolda MP, Goodhue RE, Dreves AJ, Lee J, Bruck DJ, Walton VM, O’Neal SD, Zalom FG (2011) *Drosophila suzukii* (Diptera: Drosophilidae): invasive pest of ripening soft fruit expanding its geographic range and damage potential. J Integr Pest Manag 2:G1–G7. 10.1603/IPM10010

[CR97] Wang C, Mas A, Esteve-Zarzoso B (2015) Interaction between Hanseniaspora uvarum and Saccharomyces cerevisiae during alcoholic fermentation. Int J Food Microbiol 206:67–74. 10.1016/j.ijfoodmicro.2015.04.022 25956738 10.1016/j.ijfoodmicro.2015.04.022

[CR98] Wickham H, Averick M, Bryan J, Chang W, McGowan LD, François R, Grolemund G, Hayes A, Henry L, Hester J, Kuhn M, Pedersen TL, Miller E, Bache SM, Müller K, Ooms J, Robinson D, Seidel DP, Spinu V, Takahashi K, Vaughan D, Wilke C, Woo K, Yutani H (2019) Welcome to the tidyverse. J Open Source Softw 4(43):1686. 10.21105/joss.01686

[CR99] Williams P, Strauss C, Wilson B, Massywestropp R (1983) Glycosides of 2-phenylethanol and benzyl alcohol in Vitis vinifera grapes. Phytochemistry 22:2039–2041. 10.1016/0031-9422(83)80040-5

[CR100] Zhang Z, Pawliszyn J (1996) Sampling volatile organic compounds using a modified solid phase microextraction device. J High Resol Chromatogr 19:155–160. 10.1002/jhrc.1240190307

