# Supplementary figures and images for: Chemical and Electrophysiological Characterisation of Headspace Volatiles from Yeasts Attractive to Drosophila suzukii
Source: J Chem Ecol. 2024 May 1;50(11):830–46. doi: 10.1007/s10886-024-01494-x (PMC11543737; doi:10.1007/s10886-024-01494-x)

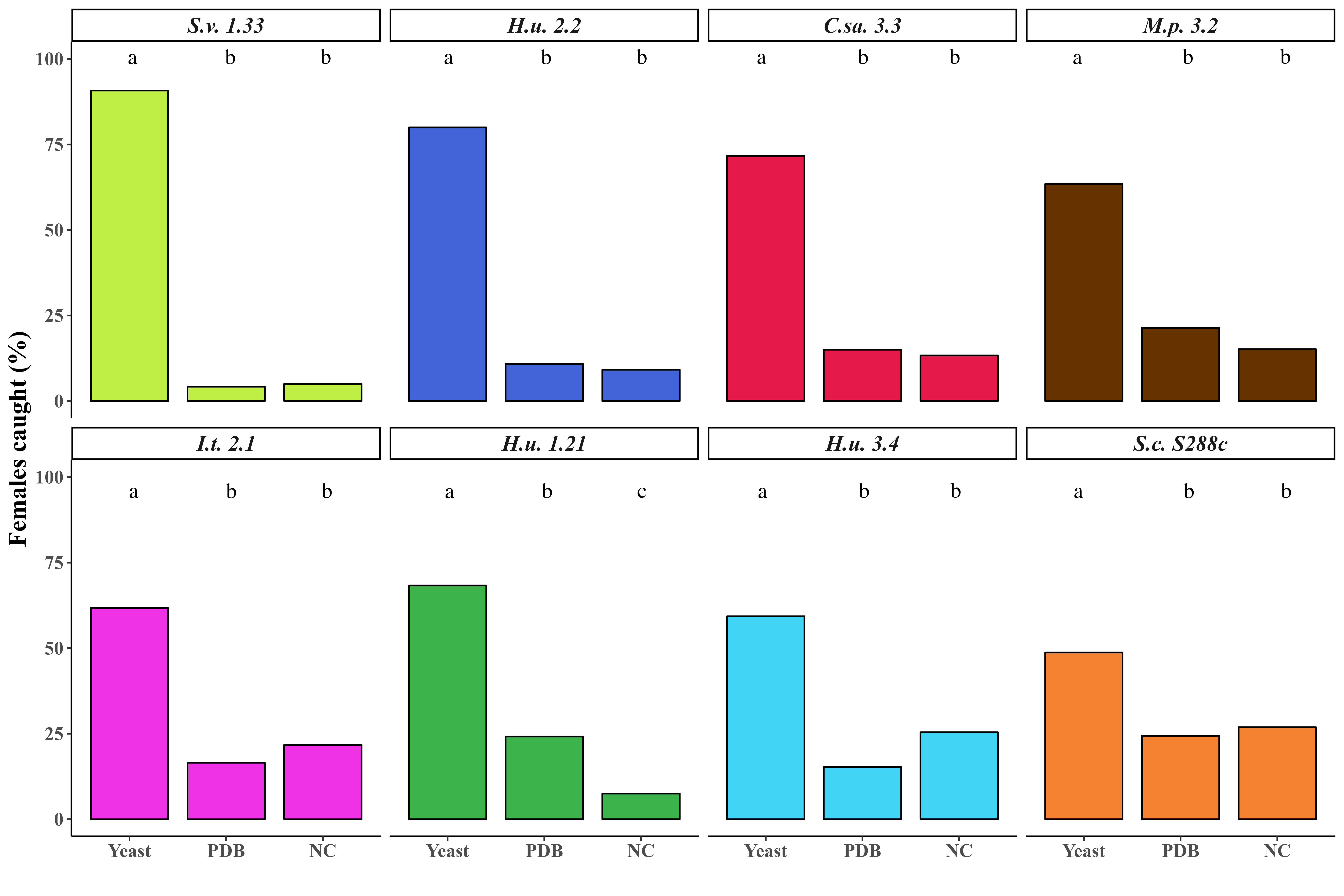

Supplement: Supplementary file 3 — Total percentage of flies trapped in liquid yeast cultures, the culture medium Potato Dextrose Broth (PDB) or not making a choice (NC) after 24 h. The bar plots show the cumulative results of six replicates (with 20 flies each) per treatment. For each yeast, different letters mean significant differences between trappings of specific yeast, PDB and NC. (GLM-Tukey contrast; p < 0.05). H.u., Hanseniaspora uvarum; S.c., Saccharomyces cerevisiae; I.t., Issatchenkia terricola; C.sa., Clavispora santaluciae; S.v., Saccharomycopsis vini; M.p., Metschnikowia pulcherrima. (JPEG 1.39 MB) [file 10886_2024_1494_MOESM3_ESM.jpeg]
